# New Coleoptera records from eastern Canada, with additions to the fauna of Manitoba, British Columbia, and Yukon Territory

**DOI:** 10.3897/zookeys.946.52489

**Published:** 2020-07-06

**Authors:** Reginald P. Webster, Pierre de Tonnancour, Jon D. Sweeney, Vincent L. Webster, Chantelle A. Kostanowicz, Cory Hughes, Robert S. Anderson, John Klymko, Claude Chantal, Robert Vigneault

**Affiliations:** 1 24 Mill Stream Dr., Charters Settlement, New Brunswick, E3C 1X1, Canada Unaffiliated Charters Settlement Canada; 2 22, 5e avenue, Terrasse-Vaudreuil, Quebec, J7V 3P5, Canada Unaffiliated Terrasse-Vaudreuil Canada; 3 Natural Resources Canada, Canadian Forest Service, Atlantic Forestry Centre, 1350 Regent St., Fredericton, New Brunswick, E3B 5P7, Canada Natural Resources Canada Fredericton Canada; 4 Canadian Museum of Nature, P.O. Box 3443, Station D, Ottawa, Ontario, K1P 6P4, Canada Canadian Museum of Nature Ottawa Canada; 5 Atlantic Canada Conservation Data Centre, P.O. Box 6416, Sackville, New Brunswick, E4L 4G7, Canada Atlantic Canada Conservation Data Sackville Canada; 6 302, rue Gabrielle-Roy, Varennes, Quebec, J3X 1L8, Canada Unaffiliated Varennes Canada; 7 16, rue du Mont-Saint-Pierre, Oka, Quebec, J0N 1E0, Canada Unaffiliated Oka Canada

**Keywords:** British Columbia, Canada, Coleoptera, Manitoba, New Brunswick, new records, Nova Scotia, Prince Edward Island, Quebec, Yukon Territory

## Abstract

One-hundred-eleven new provincial and territorial Coleoptera records are reported from New Brunswick (64), Nova Scotia (20), Prince Edward Island (5), Quebec (14), Manitoba (3), British Columbia (3), and Yukon Territory (2) for the 26 following families: Carabidae, Dytiscidae, Histeridae, Staphylinidae, Scarabaeidae, Buprestidae, Eucnemidae, Elateridae, Cantharidae, Erotylidae, Monotomidae, Cryptophagidae, Passandridae (first record of this family from New Brunswick), Laemophloeidae, Nitidulidae, Anamorphidae, Coccinellidae, Latridiidae, Mordellidae, Tenebrionidae, Cerambycidae, Chrysomelidae, Anthribidae, Brentidae, Dryophthoridae, and Curculionidae. Among these are ten new Canadian records: *Heterosternutaoppositus* (Say, 1823) (Dytiscidae) (New Brunswick), *Gyrophaenablatchleyi* Seevers, 1951 (Staphylinidae) (Quebec), *Acropteroxyslecontei* Crotch, 1873 (Erotylidae) (Manitoba), *Placonotusfalinorum* Thomas, 2011 (Laemophloeidae) (Quebec), *Adelina pallida* (Say, 1824) (Tenebrionidae) (Quebec), *Poeciloceraharrisii* (J.L. LeConte, 1851) (Chrysomelidae) (New Brunswick), *Plesiobarisalbilata* (LeConte, 1876) (Curculionidae) (Quebec, New Brunswick), *Pseudopityophthorusasperulus* (LeConte, 1868) (Curculionidae) (Nova Scotia), *Hylurgopspalliatus* (Gyllenhal, 1813) (Curculionidae) (New Brunswick), and *Heteroboripsseriatus* (Blandford, 1894) (Curculionidae) (Nova Scotia). *Plesiobarisdisjuncta* Casey reported as new for Canada in New Brunswick and Quebec by Webster et al. (2012a) is actually *P.albilata* (LeConte) and thus *P.disjuncta* is removed from the faunal list of Canada. Eleven species from New Brunswick not previously reported in literature were found on the online platforms BugGuide.Net and iNaturalist and are reported in this publication. This highlights the importance of online platforms dedicated to recording wildlife observations and citizen science in detecting new species records. Data is also presented for seven species from Quebec and two species from New Brunswick reported by Bousquet et al. (2013) without any supporting information for their occurrence in these provinces. Among the species reported here, 32 are adventive.

## Introduction

The Coleoptera of New Brunswick, Nova Scotia, and Quebec have received considerable attention since the publication of Bousquet et al. (1991) as shown by the significant increase in the number of known species from these three provinces in Table 1 in Bousquet et al. (2013). Another 303 species were added to the New Brunswick provincial list in “The Coleoptera of New Brunswick and Canada: Providing Baseline Biodiversity and Natural History Data” (Bouchard et al. 2016). Webster (2016) included an updated checklist for New Brunswick in this special issue of ZooKeys and reported 3,062 species. Additional species of Staphylinidae were reported from New Brunswick by Klimaszewski et al. (2017, 2018a, 2018b) (Aleocharinae) and Knopf and Gilmore (2018) (Staphylininae). A new coccinellid was added to the faunal list by McAlpine et al. (2018). The checklist by Bousquet et al. (2013) included 2,286 species for Nova Scotia and 4,127 for Quebec. Since then, many additional Coleoptera species have been reported from Quebec in a number of recent publications: Van Vondel and Alarie (2016) (Haliplidae), Brousseau et al. (2014) (Histeridae), Klimaszewski et al. (2018a, 2018b) (Staphylinidae, Aleocharinae), Sikes et al. (2016) (Silphidae), Hardy (2014) (Scarabaeidae), Jendek et al. (2015) (Buprestidae), Pelletier and Hébert (2014) (Cantharidae), Háva and Nei (2016) (Dermestidae), Lebel et al. (2019) (Cleridae), Bousquet and Bouchard (2014) and Steiner (2016) (Tenebrionidae), Bousquet et al. (2017) and Wappes and Santos-Silva (2019) (Cerambycidae), Barney et al. (2013) (Chrysomelidae), and de Tonnancour et al. (2017) and Dumont and de Tonnancour (2019) (Curculionoidea). A recent paper by Pentinsaari et al. (2019) using DNA barcoding added other new records from various families to all three provinces. Pelletier and Hébert (2019) reported many new Cryptophagidae (mostly *Atomaria*) to the above provincial faunal lists.

In this publication we report new records from New Brunswick, Nova Scotia, Prince Edward Island, Quebec, Manitoba, British Columbia, and Yukon Territory, including ten new Canadian records.

## Methods and conventions

### Collection methods

Various methods such as treading, sifting litter, hand collecting, and sweeping foliage were employed to collect the specimens reported in this publication. Details are outlined in Webster et al. (2009, appendix). Some specimens were collected from Lindgren funnel trap samples during a study to develop improved methods for detection of invasive species of Cerambycidae. These traps are visually similar to tree trunks and are often effective for sampling species of Coleoptera that live in microhabitats associated with standing trees (Lindgren 1983). Traps were baited with various combinations of lures used for detecting Cerambycidae and Scolytinae. See Hughes et al. (2014) and Webster et al. (2016a) for details of the lures used and methods used to deploy Lindgren traps and sample collection. Most specimens from Quebec were either swept or beaten from various plant species, attracted to mercury vapor, ultraviolet or porch lights or handpicked from various substrates or from a flight interception trap (window or tulle fabric). A description of the habitat was recorded for many specimens reported in this survey. Locality and habitat data are presented as written on the labels for each record except for habitat and collection method data recorded in French which were translated to English for Quebec records. Information is separated by a // in the data presented from each specimen where more than one label is present. GPS data are presented in decimal degrees.

### Specimen preparation and determination

Males of some species were dissected to confirm their identities. The genital structures were dehydrated in absolute alcohol and either mounted in Canada balsam on celluloid microslides or glued onto cards that were then pinned with the specimen from which they originated. Most specimens reported in this study were determined by the authors by examination and comparison of specimens in the collections at the CNC and CMNC (Curculionidea) and using various keys such as Bousquet (2010) for Carabidae, Ashbee et al. (2017) for Haliplidae, Larsin (2000) for Dytiscidae and many other keys to families or genera in other publications cited in the species accounts in this publication. Species that could not be confidently determined were sent to experts at the CNC: Adam Brunke (Staphylinidae: Aleocharinae and Oxytelinae), Patrice Bouchard (Tenebrionidae), Anthony Davies (Nitidulidae, Staphylinidae, Paederinae), Hume Douglas (Elateridae, Chrysomelidae), Serge Laplante (Histeridae, Elateridae, Erotylidae, Laemophloeidae), and Karine Savard (Chrysomelidae). Other experts consulted were Donald Bright (Scolytinae, *Pityophthorus*) and the late Michael C. Thomas (Laemophloeidae).

### Internet records

A number of species records from New Brunswick not previously recorded in Bousquet et al. (2013), Webster (2016), or other recent publications were found on BugGuide.Net and iNaturalist. Many are based on photographs of living adults and specimen vouchers are not available for further study. Only species for which determination could be confirmed by experts are reported in this paper. These records are reported in **bold** for New Brunswick (see below) under the **Distribution in Canada and Alaska** in order to note their presence in New Brunswick but are treated as previously reported records for the province by those who submitted them.

### Distribution

All species are cited with current Distribution in Canada and Alaska, using abbreviations for the states, provinces, and territories. New provincial records are indicated in **bold** under the heading Distribution in Canada and Alaska. The following abbreviations are used in the text:

**AK** Alaska

**MB** Manitoba

**YT** Yukon Territory

**ON** Ontario

**NT** Northwest Territories

**QC** Quebec

**NU** Nunavut

**NB** New Brunswick

**BC** British Columbia

**PE** Prince Edward Island

**AB** Alberta

**NS** Nova Scotia

**SK** Saskatchewan

**NL & LB** Newfoundland and Labrador^*^

USA state abbreviations follow those of the US Postal Service.

Acronyms of collections referred to in this study are as follows:

**AFC** Atlantic Forestry Centre, Fredericton, New Brunswick, Canada

**CCC** Claude Chantal Collection (private collection), Varennes, Quebec, Canada

**CFIADC** Canadian Food Inspection Agency Diagnostic Collection, Ottawa, Ontario, Canada

**CMNC** Canadian Museum of Nature Collection, Ottawa, Ontario, Canada

**CNC** Canadian National Collection of Insects, Arachnids, and Nematodes, Agriculture and Agri-Food Canada Research Centre, Ottawa, Ontario, Canada

**CTC** Claude Tessier Collection (private collection), Quebec, Quebec, Canada

**NBM** New Brunswick Museum, Saint John, New Brunswick, Canada

**PdTC** Pierre de Tonnancour Collection (private collection), Terrasse-Vaudreuil, Quebec, Canada

**RWC** Reginald Webster Collection (private collection), Charters Settlement, New Brunswick, Canada

**RVC** Robert Vigneault Collection (private collection), Oka, Quebec, Canada

**SDC** Stéphane Dumont Collection (private collection), Montreal, Quebec, Canada

## Results

One-hundred-eleven new provincial and territorial Coleoptera records are reported from NB (64), NS (20), PE (5), QC (14), MB (3) BC (3), and YT (2) from 26 families. Among these are ten new Canadian records. Eleven species from NB not included in published checklists or publications were found on BugGuide.Net and iNaturalist and are reported in this publication. Data is presented for seven species from QC and two species from NB reported by Bousquet et al (2013) without any supporting information for their occurrence in these provinces. We also remove *Plesiobarisdisjuncta* Casey (Curculionidae) from the Canadian list (Bousquet et al. 2013) based on incorrectly identified specimens of *Plesiobarisalbilata* (LeConte) from QC and NB (Webster et al. 2012a). Among the species reported here, 32 are adventive.

### Species accounts

Species which are adventive to Canada are indicated with †, Holarctic species with *. The determination that a species record was new is based on absence from print version of Bousquet et al. (2013), Webster (2016), and other publications since Bousquet et al. (2013). The classification used below follows Bousquet et al. (2013), except for the Aleocharinae (Staphylinidae) which follows Klimaszewski et al. (2018b).

#### Family Carabidae Latreille, 1802

**Subfamily Cicindelinae Latreille, 1802**

***Cicindelascutellaris* Say, 1823, new to New Brunswick**

**Note.** The closest known localities for this tiger beetle are in central ME in Clinton, Fairfield, and Skowhegan (Dearborn et al. 2014). The NB population appears to be very small; in addition to the four individuals that were captured on May 20, 2018, only 10 individuals were observed, including a mating pair. Six individuals were observed on May 25, 2018 at the same site, including another mating pair. This tiger beetle was not observed in adjacent open sandy areas with more coarse sand. The sand blowout with the fine sand where the specimens were observed is quite small (only a few hectares). Additional surveys should be conducted in open sandy areas in NB and adjacent parts of ME to see if this species is more widespread and occurs in intervening areas between the known populations. The subspecies in NB is *C.s.lecontei* Haldeman, 1853.

**Specimen data. New Brunswick, York Co.**, Upper Brockway, 20.V.2018, R.P. Webster // Jack pine forest, large bare sand area (fine sand) (2, NBM, 2, RWC).

**Distribution in Canada and Alaska.**MB, ON, QC, **NB** (Bousquet et al. 2013).

***Cicindelatranquebarica* Herbst, 1806, new to Yukon Territory**

**Note.** This species has a wide distribution across Canada from NF to NT (Bousquet et al. 2013) and its presence in YT was not unexpected. Adults were very common on moist clay along a trail through a native grassland area. The subspecies occurring in YT is likely *C.t.kirbyi* LeConte, 1867 which ranges east to MB (Bousquet et al. 2013).

**Specimen data. Yukon Territory**, 18 km N jct. Rtes. 1 & 2, W of Rt. 2, grassland area with poplar stands, 60.9571N, 135.1752W, 22.V.2016, R.P. Webster & M.-A. Giguère (8, RWC).

**Distribution in Canada and Alaska. YT**, NT, AB, SK, MB, ON, QC, NB, NS, PE, LB, NF (Bousquet et al. 2013).

#### Subfamily Harpalinae Bonelli, 1810

***Chlaeniustomentosus* (Say, 1823), new to New Brunswick**

**Note.** This species was known to range as far northeast as Quebec City in Canada and to southwestern ME in the United States (Dearborn et al. 2014). The record from NB represents a significant range extension to the northeast. This xerophilous species is often found in gravel pits and other xeric habitats (Bousquet 2010, Dearborn et al. 2014). It is likely more widespread in the region and should be searched for in sandy dry habitats.

**Specimen data. New Brunswick, York Co.**, Charters Settlement, 45.8395N, 66.7391W, residential area, on driveway, 2.IX.2017, R.P. Webster (1, RWC).

**Distribution in Canada and Alaska.**AB, SK, MB, ON, QC, **NB** (Bousquet et al. 2013).

***Acupalpuspumilus* Lindroth, 1968, new to New Brunswick**

**Specimen data: New Brunswick, Queens Co.**, Scotchtown, Grand Lake Meadows P.N.A. (Protected Natural Area), 45.8763N, 66.1822W, 16.VI.2013, R.P. Webster // Lakeshore / sand dune with red oak, sifting flood debris (1, RWC). **York Co.**, 8.5 km W of Tracy off Rte. 645, 45.6821N, 66.7894W, 6.V.2008, R.P. Webster, coll. // Wet alder swamp in leaf litter & grass on hummocks (1, NBM); Spednic Lake P.N.A. near Diggity Stream (and Pats Brook), 45.6210N, 67.4342W, 15.VI.2018 (4), 16.VI.2018 (2), R.P. Webster // Freshwater marsh, treading *Carex* hummocks (2, NBM; 4, RWC); Spednic Lake Prov. Park, 45.6183N, 67.4276W, 20.VI.2018, R.P. Webster // Marsh near Diggity Stream, treading *Carex* & grass into water (11, NBM; 6, RWC).

**Distribution in Canada and Alaska.**ON, QC, **NB**, NS, PE (Bousquet et al. 2013).

***Anisodactylusmerula* (Germar, 1824), new to New Brunswick**

**Note.** This carabid is widespread in southern ME with one isolated record from Columbia Falls in Washington Co. in eastern ME (Dearborn et al. 2014).

**Specimen data. New Brunswick, York Co.**, Spednic Lake P.N.A., trail S of East Brook Rd., 45.6716N, 67.4576W, 21.VI.2018, R.P. Webster // Sand pit, under leaves on sand (1, NBM; 1, RWC).

**Distribution in Canada and Alaska.**MB, ON, QC, **NB** (Bousquet et al. 2013).

***Stenolophushumidus* Hamilton, 1893, new to New Brunswick**

**Specimen data. New Brunswick, York Co.**, Spednic Lake P.N.A., East Brook Rd., 45.6745N, 67.4605W, 21.VI.2018, R.P. Webster // Brook with marshy margin, treading vegetation (1, RWC).

**Distribution in Canada and Alaska.**ON, QC, **NB**, NS (Bousquet et al. 2013).

***Cymindisplaticollis* (Say, 1823), new to New Brunswick**

**Note.***Cymindisplaticollis* has been recorded as far north as southern QC at Mt. St. Gregoire and Iberville, and in southern ME at Appleton (Dearborn et al. 2014). The records from NB are a significant range extension to the northeast. Most specimens were captured in Lindgren funnel traps deployed in tree canopies in mixed forests.

**Specimen data. New Brunswick, Queens Co.**, C.F.B. Gagetown, 45.7516N, 66.1866W, 18.VII–31.VIII.2018, C. Alderson & V. Webster // Old mixed forest with *Quercusrubra*, Lindgren funnel trap 1 m high under trees (1, AFC). **York Co.**, Spednic Lake P.N.A., 45.6751N, 67.4726W, 24.V–6.VI.2018 (3), 21.VI–4.VII.2018 (1), 31.VII–16.VIII.2018 (1), 16–30.VIII.2018 (4), 30.VIII–12.IX.2018 (3), C. Alderson & V. Webster // Mixed forest, Lindgren funnel traps in tree canopies (2, AFC; 3, NBM; 7, RWC).

**Distribution in Canada and Alaska.**ON, QC, **NB** (Bousquet et al. 2013).

***Colliurispensylvanica* (Linnaeus, 1758), new to New Brunswick**

**Specimen data. New Brunswick, York Co.**, Charters Settlement, 45.8395N, 66.7391W, 3.VII.2016, R.P. Webster // Residential lawn, in grass (1, RWC).

**Distribution in Canada and Alaska.**ON, QC, **NB** (Bousquet et al. 2013).

*Pterostichusbrevicornis* (Kirby, 1837)*, new to New Brunswick

**Specimen data. New Brunswick, Restigouche Co.**, Mount Atkinson, 447 m elev., 47.8192N, 68.2618W, 21.VII.2010, M. Turgeon & R.P. Webster // Boreal forest, small shaded spring-fed brook with mossy margin, sifting moss (1, RWC).

**Distribution in Canada and Alaska.**AK, YT, NT, NU, BC, AB, MB, ON, QC, **NB**, LB, NF (Bousquet et al. 2013).

#### Family Haliplidae Aubé, 1836

***Haliplusapostolicus* Wallis, 1933, new to New Brunswick**

**Specimen data. New Brunswick, Sunbury Co.**, Sand Brook Rd. at Sand Brook, 45.4984N, 66.6014W, 18.IX.2017, R.P. Webster // Stream margin in dense trailing vegetation in embayment (1, RWC).

**Distribution in Canada and Alaska.**QC, **NB**, NS (Bousquet et al. 2013).

#### Family Dytiscidae Leach, 1815

**Subfamily Hydroporinae Aubé, 1836**

***Heterosternutaoppositus* (Say, 1823), new to Canada and New Brunswick**

**Note.**Matta and Wolfe (1981) included this species as occurring in eastern Canada but did not provide any supporting data. Larson et al. (2000) did not see Canadian specimens and Bousquet et al. (2013) did not include it as a member of the Canadian fauna. Here, we provide supporting data for its occurrence in Canada.

**Specimen data. New Brunswick, Restigouche Co.**, Jacquet River Gorge P.N.A., 47.8010N, 66.0962W, 15.VIII.2010, R.P. Webster // Margin of Jacquet River, backwater pool with gravel/clay bottom (1, RWC).

**Distribution in Canada and Alaska. NB**

*Hydroporusmorio* Aubé, 1838*, new to New Brunswick

**Specimen data. New Brunswick, Carleton Co.**, Juniper Barrens at Juniper Station, 46.5534N, 67.1847W, 21.VI.2005, R.P. Webster, coll. // Black spruce bog, shaded moss-lined pool (1, RWC). **York Co**., 14 km WSW of Tracy, S of Rt. 645, 45.6741N, 66.8861W, 10–26.V.2010, R. Webster & C. MacKay, coll. // Old mixed forest with red & white spruce, red & white pine, balsam fir, eastern white cedar, and *Populus* sp., Lindgren funnel trap (1, RWC).

**Distribution in Canada and Alaska.**AK, YT, NT, NU, BC, AB, SK, MB, ON, QC, **NB**, NS, LB, NF (Bousquet et al. 2013).

***Sanfilippodytesplaniusculus* (Fall, 1923), new to New Brunswick**

**Specimen data. New Brunswick, Restigouche Co.**, 1.5 km S of Quebec (border), 425 m elev., 47.9058N, 68.1505W, 22.VI.2010, R.P. Webster, coll. // Boreal forest, small cold shaded brook, in gravel in brook (1, RWC).

**Distribution in Canada and Alaska.**QC, **NB**, LB, NF (Bousquet et al. 2013).

#### Family Histeridae Gyllenhal, 1808

**Subfamily Dendrophilinae Reitter, 1909**

***Paromalusseeversi* (Wenzel, 1936), new supporting data for Quebec**

**Note.** The first Canadian record of *Paromalusseeversi* was based on a single collection made in 1967, in Essex Co., ON (Bousquet and Laplante 2006). Included by Bousquet and Laplante (2000) in a list of species that may eventually be found in QC, this species was later recorded from this province by Bousquet et al. (2013) based on four specimens (reported below) from Terrasse-Vaudreuil collected by P. de Tonnancour, in 2013. Subsequently, numerous additional individuals were found in moist organic debris samples extracted from two hollow trees [silver maple (*Acersaccharinum* L.) and American linden (*Tiliaamericana* L.)] in a suburban residential area. This microhabitat matches closely the description given by Kovarik and Caterino (2001). Under-sampling undoubtedly explains the rarity of this species and other cavity dwelling histerids in collections.

**Specimen data. Quebec, MRC de Vaudreuil-Soulanges**, Terrasse-Vaudreuil, 45.3924N, 73.9921W, 3.VII.2013 (4), 16.VIII.2018 (1), 19.VIII.2018 (3), 22.VIII.2018 (1), 23.VIII.2018 (14), 27.VIII.2018 (12), 4.IX.2018 (6), 5.IX.2018 (8), 19.VII.2019 (8), P. de Tonnancour, moist organic debris (rotten *Cerioporussquamosus*) extracted from a tree cavity of an old *Acersaccharinum* (2, CCC; 4, CMNC; 4, CNC; 37, PdTC; 8, RVC; 2, SDC); same collector but Terrasse-Vaudreuil, 45.3926N, 73.9929W, 9.VII.2019, moist organic debris extracted from a hollow trunk section of a large *Tiliaamericana* occupied by red squirrels, 8 m above ground (17, PdTC).

**Distribution in Canada and Alaska.**ON, QC (Bousquet et al. 2013).

#### Family Staphylinidae Latreille, 1802

**Subfamily Pselaphinae Latreille, 1802**

***Euplectuskarstenii* (Reichenbach, 1816) †, new to New Brunswick**

**Note.**Wagner (1975) reported this adventive species from well-rotted haystacks, corncob piles, horse manure, and occasionally from tree holes. The two NB specimens were captured in Lindgren funnel traps in conifer forests.

**Specimen data. New Brunswick, Sunbury Co.**, Acadia Research Forest, 45.9868N, 66.3841W, 13–21.VII.2009, R.P. Webster & M.-A. Giguère, coll. // Red spruce forest with red maple & balsam fir, Lindgren funnel trap (1 ♀♂ (dissected), RWC). **York Co.**, Fredericton, Odell Park, 45.9571N, 66.6650W, 15–28.VI.2012, C. Alderson & V. Webster // Old-growth hemlock forest, Lindgren funnel trap in canopy of *Betulaalleghaniensis* (1 ♂ (dissected), RWC).

**Distribution in Canada and Alaska.**BC, SK, MB, ON, QC, **NB**, PE (Bousquet et al. 2013).

**Subfamily Aleocharinae Fleming, 1821**

***Philhygrapseudopolaris* Klimaszewski and Langor, 2011, new to British Columbia**

**Note.** The BC specimens were collected by treading emergent grasses and sedges in a small pond. Nothing was previously known about the habitat associations of this species (Klimaszewski et al. 2018b).

**Specimen data. British Columbia**, Rt. 97 near Smart River, 59.9326N, 131.7556W, 6.VI.2019, R.P. Webster // Small pond with emergent grasses & sedges (2 ♂♂ (dissected), 3 ♀♀ (dissected), RWC).

**Distribution in Canada and Alaska.**AK, YT, NT, **BC**, MB, QC, NF (Klimaszewski et al. 2018b).

***Gyrophaenablatchleyi* Seevers, 1951, new to Canada and Quebec**

**Note.** This species has been reported from MI and IN in the United States (Seevers 1978; Enushchenko 2017). The record reported here is the first for Canada and represents a significant range extension to the northeast. The initial determination made by Tim Struyve was confirmed by Adam Brunke.

**Specimen data: Canada, Quebec, MRC de Deux-Montagnes**, Oka, parc national d’Oka, Calvaire d’Oka, 25.VI.2016, R. Vigneault (1 ♂ (dissected), RVC).

**Distribution in Canada and Alaska. QC**.

***Caloderaparviceps* (Casey, 1893) new to British Columbia**

**Specimen data. British Columbia**, Rt. 97 near Smart River, 59.9326N, 131.7556W, 6.VI.2019, R.P. Webster // Small pond with emergent grasses & sedges (1 ♀ (dissected), RWC).

**Distribution in Canada and Alaska.**YT, **BC**, ON, NB, NS (Klimaszewski et al. 2018b).

***Phloeoporacanadensis* Klimaszewski and Langor, 2011, new to Nova Scotia**

**Specimen data. Nova Scotia, Annapolis Co.**, Kejimkujik N.P., 44.40366N, 65.21969W, 13–30.VIII.2018, Elyse Simms, coll. // Mixed forest, Lindgren funnel trap, Trap 3 (1, AFC). **Queens Co.**, Kejimkujik N.P., 44.38505N, 65.20715W, 30.VII–13.VIII.2018, G. Marten-Carpenter, coll. // Mixed forest, Lindgren funnel trap, Trap 10 (1, AFC).

**Distribution in Canada and Alaska.**BC (CNC, Klimaszewski et al. 2020), NB, **NS**, NF (Klimaszewski et al. 2018b).

**Subfamily Oxytelinae Fleming, 1821**

***Anotylussuspectus* (Casey, 1893), new to New Brunswick**

**Note.** All specimens were sifted from material from a large nest of a black *Formica* ant species. R. P. Webster has not found this species elsewhere in NB in ant nests. It is unclear if this species is normally associated with ants.

**Specimen data. New Brunswick, Carleton Co.**, Meduxnekeag Valley Nature Preserve, 46.1979N, 67.6854W, 21.V.2005, M.-A. Giguère & R.P. Webster, coll. // Mixed forest, in large nest of black *Formica* species (8, RWC), same data as above but 4.V.2008, R.P. Webster, coll. (2, RWC).

**Distribution in Canada and Alaska.**MB, ON, **NB** (Bousquet et al. 2013).

***Anotylustetracarinatus* (Block, 1799) †, new to New Brunswick**

**Note.** The adventive *Anotylustetracarinatus* was newly reported from NB by Webster et al. (2012e). Re-examination of these specimens revealed that they are a different species, tentatively determined by Adam Brunke as *A. nanus* Erichson, 1840. Until a proper review of the native species is completed this determination is considered tentative (Brunke, pers. com.). One specimen of *A. tetracarinatus* (determined by Adam Brunke and reported below) was recently collected from compost, maintaining this species on the faunal list of NB.

**Specimen data. New Brunswick, York Co.**, Charters Settlement, 45.8395N, 66.7391W, 17.V.2018, R.P. Webster // Mixed forest, in decaying corncobs & cornhusks (1 ♂ (dissected), RWC).

**Distribution in Canada and Alaska.**BC, AB, ON, QC, **NB**, NS (Bousquet et al. 2013).

***Carpelimuserichsoni* (Sharp, 1871) †, new to Quebec**

**Note.** This adventive species is listed by Schülke and Smetana (2015) as widely distributed in southern Europe from Russia (Southern Territory) and Yugoslavia south to Algeria and east to the Netherlands and Belgium. It was recently reported from North America from Charters Settlement, NB by Webster et al. (2016b). The specimen reported below was collected at light. The initial determination made by Tim Struyve was confirmed by Adam Brunke.

**Specimen data. Quebec, MRC de Deux-Montagnes**, Oka, parc national d’Oka, 19.VI.2016, R. Vigneault, deciduous forest near beach, attracted to mercury vapor lamp (1 ♂ (dissected), RVC).

**Distribution in Canada and Alaska. QC**, NB (Webster et al. 2016b).

***Carpelimusgracilis* (Mannerheim, 1830) †, new to Quebec**

**Note.** This adventive species has long been known to occur in North America (Fauvel 1889) but was reported only recently from Canada, in NB (Webster et al. 2016b). The specimen recorded below was collected with a car net along a road running through a deciduous forest. The initial determination made by Tim Struyve was confirmed by Adam Brunke.

**Specimen data. Quebec, MRC de Deux-Montagnes**, Oka, parc national d’Oka, chemin des Collines, 12.VII.2016, T. Struyve, car net (1 ♂ (dissected), RVC).

**Distribution in Canada and Alaska. QC**, NB (Webster et al. 2016b).

***Oxytelusnimius* Casey, 1893, new to New Brunswick**

**Note.** Most NB specimens were collected from horse or deer dung. One was found among debris in the entrance of a woodchuck or groundhog [*Marmotamonax* (L.)] burrow.

**Specimen data. New Brunswick, York Co.**, Keswick Ridge, 45.9962N, 66.8781W, 25.V.2015, R.P. Webster // Old field / forest margin, entrance to *Marmotamonax* (L.) burrow (1, RWC); Charters Settlement, 45.8349N, 66.7436W, 8.V.2018, R.P. Webster // Old brushy field, in horse dung on gravel road (1, RWC); same locality but, 45.8447N, 66.7292W, 28.V.2018, R.P. Webster // Mixed forest in deer dung (5, RWC).

**Distribution in Canada and Alaska.**ON, QC, **NB** (Bousquet et al. 2013).

**Subfamily Steninae MacLeay, 1825**

***Stenusquebecensis* Puthz, 1971, new to Yukon Territory**

**Note.** This species has a wide distribution from NF to AK (Bousquet et al. 2013) and its presence in YT was not unexpected. The two specimens were collected from emergent sedges in a gravel pit pond.

**Specimen data. Yukon Territory**, 47 km W of Watson Lake off Rt.1, 60.1313N, 129.5523W, 5.VI.2019, R.P. Webster // Gravel pit pond with emergent sedges (1 ♂, 1 ♀ (dissected), RWC).

**Distribution in Canada and Alaska.**AK, **YT**, NT, BC, AB, SK, MB, ON, QC, NB, NS, NF (Bousquet et al. 2013).

*Stenusniveus* Fauvel, 1865*, new to British Columbia, new supporting data for New Brunswick

**Note.***Stenusniveus* was reported from NB by Bousquet et al. (2013) on the basis of the record below. This species is reported for the first time from BC. Adults were tread from *Sphagnum* and *Carex* hummocks in NB and emergent grasses and *Carex* on the margin of a pond in BC.

**Specimen data. New Brunswick, Charlotte Co.**, near New River, 7.VII. 2006, 13.VI.2008, 45.2118N, 66.6179W, R.P. Webster, coll. // Small marsh, treading *Sphagnum* & *Carex* hummocks into water (3 ♂, 1 ♀ (dissected), RWC).

**British Columbia**, Rt. 97 near Smart River, 59.9326N, 131.7556W, 6.VI.2019, R.P. Webster // Small pond with emergent grasses & sedges (1 ♀ (dissected), RWC).

**Distribution in Canada and Alaska.**AK, YT, NT, **BC**, MB, NB (Bousquet et al. 2013).

**Subfamily Paederinae Fleming, 1821**

***Pseudolathraambigua* (LeConte, 1880), new to New Brunswick**

**Specimen data. New Brunswick, York Co.**, Douglas, Currie Mountain, 45.9844N, 66.7592W, 19.VIII–6.IX.2013, C. Hughes & V. Webster // Mixed forest with *Quercusrubra*, Lindgren funnel trap in canopy of *Q.rubra* (1 ♂ (dissected), RWC).

**Distribution in Canada and Alaska.**ON, QC, **NB** (Bousquet et al. 2013).

***Tetartopeustetricus* Casey, 1905, new to New Brunswick**

**Note.**Watrous (1980) reported *Tetartopeustetricus* from NH and VT but it has not yet been reported from ME (Majka et al. 2011). Watrous (1980) notes that *Tetartopeus* species are usually found in wetland habitats and occur among damp leaf litter, moss, and other debris along streams, marshes, bogs, ponds, including vernal ponds in swamps and forests, but he does not provide any details of the microhabitat associations for *T.tetricus*. Most NB specimens of *T.tetricus* were found among moist leaves on the margin of a vernal pond in a mixed forest. The first author has found this species in very similar habitats in RI.

**Specimen data. New Brunswick, York Co.**, Spednic Lake P.N.A., East Brook Rd., 45.6752N, 67.4739W, 9.V.2018 (3), 21.VI.2018 (1), R.P. Webster // Mixed forest, vernal pond margin in leaf litter (1 ♀, NBM; 1 ♂, 2 ♀♀ (dissected), RWC); Spednic Lake P.N.A., 45.6980N, 67.4982W, 12.VI.2018, R.P. Webster & M.-A. Giguère // Stream margin, in gravel / sand (1 ♂, NBM).

**Distribution in Canada and Alaska.**ON, QC, **NB** (Bousquet et al. 2013).

**Subfamily Staphylininae Latreille, 1802**

***Erichsoniuscivicus* Frank, 1975, new to New Brunswick**

**Note.***Erichsoniuscivicus* has been recorded from NH and NY south to GA and LA (Frank 1975) and FL (Frank 1981) but has not yet been recorded from ME (Majka et al. 2011). Specimens reported below were collected from bare mud and treading vegetation in a marsh along the margin of a slow flowing stream. One individual was collected from moist leaves on a vernal pond margin, another was captured in a Lindgren funnel trap adjacent to a marsh. This species was collected from May to August.

**Specimen data. New Brunswick, York Co.**, Dumfries, Slagundy Dry Ponds, 45.85960N, 67.18490W, 8.VII.2008, R.P. Webster, coll. // Large vernal pond, in moist leaves near water (1 ♂ (dissected), NBM); Spednic Lake P.N.A., near Diggity Stream, 45.6205N, 67.4319W, 10.VIII.2017, R.P. Webster // Marsh near slow flowing stream, on bare mud (5) and treading vegetation (2) (5 ♂♂ , 2 ♀♀ (dissected), RWC); Spednic Lake P.N.A., near Pats Brook, 45.6210N, 67.4342W, 16.VI.2018, R.P. Webster // Freshwater marsh with slow flowing stream with emergent vegetation, treading vegetation (1, RWC); Spednic Lake P.N.A., Palfrey Stream at East Brook Rd., 45.6988N, 67.4987W, 21.VIII.2017, R.P. Webster & M.-A. Giguère // Rocky to gravel stream margin, under rock near alders (1 ♂ (dissected), NBM); Spednic Lake P.N.A., Small stream at East Brook Rd., 45.6982N, 67.4969W, 20.VI.2018, R.P. Webster // Brook with marshy margin, treading vegetation (1, RWC); Spednic Lake P.N.A., 45.6201N, 67.4297W, 10–24.V.2018, C. Alderson & V. Webster // Marsh margin near mixed forest, Lindgren funnel trap 1 m above ground on red maple (1 ♂ (dissected), NBM).

**Distribution in Canada and Alaska.**ON, QC, **NB** (Bousquet et al. 2013).

***Hesperusapicialis* (Say, 1830), new to Nova Scotia**

**Specimen data. Nova Scotia, Annapolis Co.**, Kejimkujik N.P., 44.39812N, 65.23401W, 30.VII–13.VIII.2018, G. Marten-Carpenter, coll. // Mixed forest, Lindgren funnel trap, Trap 7 (1, AFC); Kejimkujik N.P., 44.40317N, 65.24613W, 30.VIII–28.X.2018, Donna Crossland, coll. // Mixed forest, Lindgren funnel trap, Trap 8 (1, AFC). **Queens Co.**, Kejimkujik N.P., 44.38505N, 65.20715W, 13–30.VIII.2018, Elyse Simms, coll. // Mixed forest, Lindgren funnel trap, Trap 10 (1, AFC).

**Distribution in Canada and Alaska.**ON, QC, NB, **NS** (Bousquet et al. 2013).

#### Family Scarabaeidae Latreille, 1802

**Subfamily Aphodiinae Leach, 1815**

***Diapternapinguis* (Haldeman, 1848)**

*Diapternapinguis* was reported for the first time from NB by Webster et al. (2012c) on the basis of a specimen from Cranberry Lake P.N.A. in Queens County. This specimen was misidentified and is *D.hyperborea* (LeConte, 1850) (Andrew Smith, pers. comm.). Andrew Smith noted that there is a NB specimen of *D.pinguis* in the CNC from Tabusintac (Northumberland County) collected by W.J. Brown on June 27, 1939, and thus maintaining the species on the NB faunal list.

**Subfamily Melolonthinae Leach, 1819**

***Sericaelusa* Dawson, 1919, new to New Brunswick**

**Specimen data. New Brunswick, York Co.**, Kingsclear, 45.9456N, 66.7948W, 29.VI–5.VII.2016 (1), 6–14.VII.2016 (3), C. Alderson & V. Webster // Mixed forest, Lindgren funnel trap 1 m above ground (4 ♂♂ (dissected), AFC); same locality and habitat data and collectors but 13.VIII.2018, white light trap (1 ♂ (dissected), NBM); Spednic Lake P.N.A., 45.6751N, 67.4726W, 6–21.VI.2018, C. Alderson & V. Webster // Mixed forest, Lindgren funnel trap in tree canopy (1 ♂ (dissected), RWC).

**Distribution in Canada and Alaska.**ON, QC, **NB** (Bousquet et al. 2013).

#### Family Buprestidae Leach, 1815

**Subfamily Agrilinae Laporte, 1835**

***Agrilusarcuatus* (Say, 1825), new to Nova Scotia**

**Note.***Agrilusarcuatus* was common at Magazine Hill with 95 individuals captured in Lindgren funnel traps during 2018. American beech (*Fagusgrandifolia* Ehrh.), a known host of this species (Paiero et al. 2012), was common at this site.

**Specimen data. Nova Scotia, Halifax Co.**, Magazine Hill, 44.7143N, 63.6331W, 1.VIII.2016, coll., K. Van Rooyen & N. Higgins (1, AFC); same locality data but 8–16.VII.2018, 16–23.VII.2018, K. Van Rooyen & J. Palmer // Hardwood dominated forest, Green Lindgren funnel trap in tree canopy (2 ♂♂ (dissected), AFC).

**Distribution in Canada and Alaska.**SK, MB, ON, QC, NB, **NS** (Bousquet et al. 2013).

***Agrilusbilineatus* (Weber, 1801), new to Nova Scotia**

**Specimen data. Nova Scotia, Halifax Co.**, Magazine Hill, 44.7143N, 63.6331W, 27.VI.2016 (1), 11.VII.2016 (1), 25.VII.2016 (1), 22.VIII.2016, coll., K. Van Rooyen & N. Higgins (4, AFC).

**Distribution in Canada and Alaska.**MB, ON, QC, NB, **NS** (Bousquet et al 2013).

***Agriluscyanescenscyanescens* (Ratzeburg, 1837)** †

This adventive buprestid species was reported for the first time from NB on BugGuide.Net by Denis A. Doucet from near the Fundy National Park Headquarters, Albert Co. based on a photo taken on July 12, 2015. See https://bugguide.net/node/view/1107551. Eduard Jendek (Canadian Food Inspection Agency) confirmed the determination.

**Distribution in Canada and Alaska.**ON, QC, **NB** (Bousquet et al. 2013).

***Agrilusegenus* Gory, 1841, new to New Brunswick**

**Note.** Black locust (*Robiniapseudoacacia* L.), which was planted at this site on mine tailings, is host of this species (Paiero et al. 2012).

**Specimen data. New Brunswick, Queens Co.**, Rt. 690 near Flowers Cove, 46.0367N, 66.0376W, 16.VI.2013, M.-A. Giguère & R.P. Webster // Roadside stand of *Robiniapseudoacacia* L., beating *Robinia* foliage (1, RWC).

**Distribution in Canada and Alaska.**ON, QC, **NB** (Bousquet et al. 2013).

***Agrilusobsoletoguttatus* Gory, 1841, new to Nova Scotia**

**Specimen data. Nova Scotia, Halifax Co.**, Magazine Hill, 44.7143N, 63.6331W, 4–11.VII.2016 (1), 2–8.VII.2018 (1), 16–23.VII.2018 (2), K. Van Rooyen & J. Palmer // Hardwood dominated forest, green Lindgren funnel trap in tree canopy (4 (2 ♂♂ dissected), AFC)

**Distribution in Canada and Alaska.**ON, QC, NB, **NS** (Bousquet et al. 2013).

***Agrilusplanipennis* Fairmaire, 1888 †, new to New Brunswick, Nova Scotia, and Manitoba**

**Note.** The emerald ash borer, *Agrilusplanipennis*, native to Asia, was first detected in North America in Detroit, MI and Windsor, ON in July 2002. As of February 2020, *A. planipennis* had been detected in 35 states in the USA (from CO to ME), and in five Canadian provinces (EAB Information Network http://www.emeraldashborer.info/about-eab.php). This invasive species has killed millions of ash trees (*Fraxinus* spp.) trees in North America; its spread has been exacerbated by human movement of infested material such as firewood (Herms and McCullough 2014). Here, we report data on its occurrence in NB, NS, and MB. Some of the new records reported below are based on larval collections from symptomatic *Fraxinus* trees and adults reared from infested *Fraxinuspennsylvanica* Marsh. bolts.

**Specimen data. Manitoba**, Winnipeg, 582 Cote Street, 49.87705N, 97.09884W, 9.XI.2017 (larval collection date), Jason Watts (2 larvae, CFIADC). **New Brunswick, Madawaska Co**., Edmundston, 633 rue St-Francois, 47.35824N, 68.360050W, 7-V-2018 (larval collection date), Bernard Michaud, coll. (16 larvae, CFIADC); same locality but 47.3580N, 68.3607W, 19.VII.2018 (adult emergence date) (*Fraxinuspennsylvanica* bolts collected May 2018), K. Van Rooyen & J. Palmer, coll. (6 adults, AFC). **Sunbury Co.**, Oromocto Highway exit, Waasis Rd. and Restigouche Road; 45.84420N, 66.510310W, 16-VII-2019, Aaron Perry, green sticky prism trap baited with Z3-hexenol + lactone (1 adult, CFIADC). **Westmoreland Co.**, Moncton, 50 Hastings Street, 46.10323N, 64.820200W, 20-VII-2019, Matt Linton, green prism trap (2 adults, CFIADC). **Nova Scotia, Halifax Co.**, Bedford, 91 Waterfront Drive, 44.71975N, 63.671530W, 7-IX-2018 (larval collection date), Ron Neville (1 larva, CFIADC); Bedford, DeWolfe Park, 44.71705N, 63.67117W, ex. *Fraxinuspennsylvanica* bolts collected Nov. 2018, Sweeney Crew (3 adults, AFC).

**Distribution in Canada and Alaska.**ON, QC, **MB, NB, NS** (Bousquet et al. 2013).

#### Family Eucnemidae Eschscholtz, 1829

**Subfamily Melasinae Fleming, 1821**

***Dirrhagofarsusernae* Otto, Muona & McClarin, 2014 †, new to Nova Scotia**

**Note.***Dirrhagofarsusernae* was newly reported from Canada and NB by Webster et al. (2016c). Although this species was described from OH, it is thought to be an introduction to North America from Asia (Otto et al. 2014). This species is widespread in the eastern United States and appears to be common in NB (Webster et al. 2016c). Its presence in NS is not unexpected.

**Specimen data. Nova Scotia, Queens Co.**, Kejimkujik N.P., 44.38505N, 65.20715W, 30.VII–13.VIII.2018, G. Marten-Carpenter, coll. // Mixed forest, Lindgren funnel trap, Trap 10 (1, AFC).

**Distribution in Canada and Alaska.**NB, **NS** (Webster et al. 2016c).

#### Family Elateridae Leach, 1815

**Subfamily Elaterinae Leach, 1815**

***Ampedusmelsheimeri* (Leng, 1918), new to New Brunswick**

**Specimen data. New Brunswick, Gloucester Co**., Bathurst, Daly Point Reserve, 47.6392N, 65.6098W, 28.V–15.VI.2015, C. Alderson & V. Webster // Mixed forest, purple Lindgren funnel trap 1 m high (1, RWC). **York Co.**, Douglas, Currie Mountain, 45.9832N, 66.7564W, 24.VI–9.VII.2013, C. Alderson & V. Webster // Old *Pinusstrobus* stand, Lindgren funnel trap in canopy of *P.strobus* (1, RWC); Fredericton, Odell Park, 29.VI–14.VII.2015 (1), 14–28.VII.2015 (2), 28.VII–10.VIII.2015 (1), C. Alderson & V. Webster // Hardwood forest, Lindgren funnel traps in canopy of hardwoods (4, RWC).

**Distribution in Canada and Alaska.**SK, MB, ON, QC, **NB**, NS (Bousquet et al. 2013).

#### Family Cantharidae Imhoff, 1856

**Subfamily Cantharinae Imhoff, 1856**

***Rhagonychahirticula* (Green, 1941), new to New Brunswick**

**Note.** Adults were swept from vegetation in freshwater marshes, a salt marsh, a calcareous fen, along the margin of a salt spring, and along a trail through a mixed forest. Two individuals were captured in Lindgren funnel traps in the canopy of trees in hardwood forests, and one from traps in a jack pine (*Pinusbanksiana* Lamb.) forest. Nothing was previously known about the habitat associations of this species (Pelletier and Hébert 2014).

**Specimen data. New Brunswick, Albert Co.**, Shepody N.W.A., Mary’s Point Section, 45.7320N, 64.6765W, 29.VI.2004, R.P. Webster, coll. // Margin of salt marsh, sweeping foliage (1, RWC). **Carleton Co.**, Jackson Falls, Bell Forest, 46.2200N, 67.7231W, 17–31.VII.2012, C. Alderson & V. Webster // Rich Appalachian hardwood forest, Lindgren funnel trap in canopy of *Tiliaamericana* (1 female, RWC). **Northumberland Co.**, ca. 2.5 km W of Sevogle, 47.0876N, 65.8613W, 26.VI–6.VII.2013, C. Alderson & V. Webster // Old *Pinusbanksiana* forest, Lindgren funnel trap (1, RWC). **Sunbury Co.**, off Coy Rd., Grand Lake Meadows P.N.A., 45.9804N, 66.1824W, 20.VI.2013, R.P. Webster / Trail through mixed forest, sweeping vegetation (alders, willows, sweet fern & blueberry) (1, RWC). **Westmorland Co**., off Fawcett Hill Rd., 45.9719N, 65.2249W, 19.VI.2012, R.P. Webster & D. Sabine // Salt spring, sweeping vegetation (1, RWC). **York Co.**, Charters Settlement, 45.8395N, 66.7391W, 20.VI.2012, 14.VII.2012, R.P. Webster // mixed forest, m.v. light (2 females, RWC); Charters Settlement, 45.8451N, 66.7289W, 17.VI.2018, R.P. Webster // Marsh below beaver dam, sweeping foliage (5, RWC); Charters Settlement, 45.8346N, 66.7328W, 17.VI.2018, R.P. Webster // Marsh / alder swamp, sweeping vegetation (2, RWC); Fredericton, Odell Park, 45.9539N, 66.6666W, 9–24.VII.2013, C. Alderson & V. Webster // Hardwood stand, Lindgren funnel trap in canopy (1, NBM); Canterbury, Eel River P.N.A., 45.8967N, 67.6343W, 23.VI.2014, R.P. Webster // Calcareous fen with shrubby cinquefoil, sweeping vegetation with *Myricagale* & Labrador tea (1, RWC).

**Distribution in Canada and Alaska.**ON, QC, **NB** (Pelletier & Hébert 2014).

#### Family Melyridae Leach, 1815

**Subfamily Malachiinae Fleming, 1821**

***Anthocomusequestris* (Fabricius, 1781)** †

This distinctive looking adventive species was reported for the first time from NB from Nasonworth, York Co. in BugGuide.Net by Eric Knopf. See https://bugguide.net/node/view/1236752. This individual was photographed on June 7, 2016. A voucher specimen was not collected.

**Distribution in Canada and Alaska.**ON, QC, **NB** (Bousquet et al. 2013).

#### Family Erotylidae Latreille, 1802

**Subfamily Loberinae Bruce, 1951**

***Loberusimpressus* LeConte, 1863, new to Quebec**

**Note.**Bousquet et al. (2013) report this species only from ON, but a photograph taken by Alain Hogue in Salaberry-de-Valleyfield, QC in 2015, 30 km north of the USA border (https://bugguide.net/node/view/1073833/bgimage), and the numerous specimens reported below confirm its presence in QC.

**Specimen data. Quebec, MRC de Deux-Montagnes**, Oka, parc national d’Oka, composting site, 45.4764N, 74.0545W, 30.V.2016, 19 h 30–20 h 30, P. de Tonnancour, flight interception trap (white tulle fabric) (2, PdTC). **MRC Le Fjord-du-Saguenay**, parc national des Monts-Valin, 48.5783N, 70.8773W, 10.VIII.2016, 21–24 h, P. de Tonnancour, attracted to mercury vapor lamp (1, PdTC). **MRC de Vaudreuil-Soulanges**, Notre-Dame-de-l’Île-Perrot, 45.3787N, 73.9426W, 19.V.2011, 14 h, P. de Tonnancour, swept from *Trifoliumrepens* (1, PdTC); Notre-Dame-de-l’Île-Perrot, 45.3766N, 73.9438W, 8.VII.2013, 15 h, P. de Tonnancour, swept from *Scirpusatrovirens* (1, PdTC); Notre-Dame-de-l’Île-Perrot, 45.3702N, 73.9592W, 13.IX.2018, 15 h, P. de Tonnancour, beaten from *Caryaovata* (1, PdTC); Notre-Dame-de-l’Île-Perrot, 45.3781N, 73.9396W, 18.VIII.2019, 13 h, P. de Tonnancour, swept from herbaceous plants (1, PdTC); Pointe-des-Cascades, parc nature de Pointe-des-Cascades, 45.3331N, 73.9557W, 1.VII.2013, 16 h, P. de Tonnancour, swept from herbaceous plants, marsh margin (4, PdTC; 2, RVC); Terrasse-Vaudreuil, 45.3923N, 73.9922W, 7.VI.2011, 22–24 h (1), 17.V.2017, 21–22 h (1), P. de Tonnancour, attracted to porch + UV lamps (2, PdTC); same locality data and collector but 21.VIII.2013, 1 h (1), 1.VIII.2018, 22–24 h (1), attracted to porch + UV + mercury vapor lamps (2, PdTC); same locality data and collector but 26.VI.2014, 23 h (1), 30.VI.2014, 23 h (1), attracted to UV lamp (2, PdTC); same locality data and collector but 6.VII.2017, 23–24 h, attracted to mercury vapor lamp (1, PdTC).

**Distribution in Canada and Alaska.**ON, **QC** (Bousquet et al. 2013).

**Subfamily Languriinae Hope, 1840**

***Acropteroxyslecontei* Crotch, 1873, new to Canada and Manitoba**

**Note.***Acropteroxyslecontei* has been reported as far north as MT and SD in the United States (Vaurie 1948, Downie & Arnett 1996). The record below is the first for Canada.

**Specimen data. Manitoba**, ca. 5 km E of jct. Hwy 21 & 345, 49.3849N, 100.4378W, 7.VII.2007, R.P. Webster, coll. // Gravel pit in short grass prairie area, sweeping vegetation, sunflowers, sweet clover, alfalfa, sage brush, etc. (5, RWC).

**Distribution in Canada and Alaska. MB.**

**Subfamily Erotylinae Latreille, 1802**

***Triplaxfestiva* Lacordaire, 1842, new to New Brunswick**

**Specimen data. New Brunswick, York Co.**, Spednic Lake P.N.A., 45.6751N, 67.4726W, 31.VII–16.VIII.2018, C. Alderson & V. Webster // Mixed forest, Lindgren funnel trap in tree canopy (1, RWC).

**Distribution in Canada and Alaska.**QC, **NB** (Bousquet et al. 2013).

**Subfamily Cryptophylinae Reitter, 1874**

***Cryptophilusobliteratus* Reitter, 1874 †, new to New Brunswick and Quebec**

**Note.***Cryptophilusseriatus* Casey, 1924 was until recently thought to be a native Nearctic species described from MA. However, Esser (2017) synonymized it with the Palaearctic *C.obliteratus* and established that it is adventive in North America. The occurrence of *C.obliteratus* in Canada, specifically in ON, was very recently confirmed by DNA barcoding (Pentinsaari et al. 2019) also representing the first record for Canada. A recent review of the 46 Canadian *Cryptophilus* specimens in the CNC initially determined as *C.integer* (Heer, 1841) revealed that all are actually *C.obliteratus* (Serge Laplante, pers. com. 2019). The vast majority were from ON, but five of them were collected in QC, in Montreal: (1.VI.1978 (1), 26.V.1982 (1), 14.VI.1986 (1), E.J. Kiteley (3, CNC); 23.IX.1987, L. LeSage (2, CNC). These specimens represent the first report of *C.obliteratus* from QC. Additional more recent specimens are reported below. This species is associated with decaying organic matter. Specimens from NB were collected in compost, and most of those from QC were found in close proximity of a regularly fed compost heap in a suburban residential area.

**Specimen data. New Brunswick, York Co.**, Charters Settlement, 45.8395N, 66.7391W, 10.V.2018 (11), 17.V.2018 (5), 17.IX.2018 (1) R.P. Webster // Mixed forest, in compost (decaying vegetable matter) (6, NBM: 11, RWC). **Quebec, Agglomération de Longueuil**, Saint-Lambert, 17.V.1970, P. de Tonnancour (1, PdTC). **MRC de Deux-Montagnes**, Oka, rue Mont-Saint-Pierre, 45.4993N, 74.0204W, 4.V.2002 (1), 16.V.2002 (3), R. Vigneault, attracted to UV lamp (4, RVC); Oka, parc national d’Oka, composting site, 45.4765N, 74.0542W, 28.IV.2013 (1), 25.V.2014 (1), R. Vigneault, flight interception trap (white tulle fabric) (2, RVC); same locality, but 3.V.2015, 14–18 h, P. de Tonnancour, flight interception trap (white tulle fabric) between log piles (white pine and deciduous) (2, PdTC). **MRC de Marguerite-D’Youville**, Varennes, 20.VI.2005 (1), 29.VI.2012 (1), 31.V.2015 (1), C. Chantal, attracted to UV lamp (3, CCC). **MRC de Vaudreuil-Soulanges**, Terrasse-Vaudreuil, 45.3923N, 73.9922W, 20.V.2014 (1), 21.V.2014, 17 h (1), 8.V.2015, 18 h (1), 21.V.2016, 13 h (2), 14.VI.2016, 18 h (1), P. de Tonnancour, flight interception trap (white tulle fabric) (6, PdTC); same locality data and collector but 28.VI.2014, 23 h, attracted to UV lamp (1, PdTC); same locality data and collector but 9.X.2018, 18–19 h, window flight interception trap baited with ill-smelling decayed plant matter (1, PdTC); Ville de L’Île-Perrot, 20.III.2013, 17 h 30 (22 °C), P. de Tonnancour, in flight (1, PdTC).

**Distribution in Canada and Alaska.**ON, **QC, NB** (Pentinsaari 2019)

***Cryptophiluspropinquus* Reitter, 1874 †, additional records for Quebec**

**Note.**Esser (2016, 2017) showed that the name *Cryptophilusinteger* (Heer, 1841) was not valid and that all North American records previously treated as *C.integer* include two adventive species, *C.propinquus* Reitter and *C.angustus* (Rosenhauer, 1856). Only *C.propinquus* was found among barcoded specimens from Canada (BC and ON) (Pentinsaari et al. 2019). *Cryptophilusinteger* was listed by Bousquet et al. (2013) from QC and ON. However, all the CNC specimens were another species, *Cryptophilusobliteratus* (see comments for previous species). *Cryptophiluspropinquus* was previously recorded from the province of QC (under the name *C.integer*) from Hemmingford, in 1933 (Klimaszewski et al., 2015). Here, we report additional modern records from the province. Most of the above specimens were collected late in the season in a suburban residential area, at most 15 m away from a regularly fed compost heap.

**Specimen data. Quebec, Agglomération de Québec**, Sainte-Foy, 30.VI.1975, C. Chantal, attracted to UV lamp (1, CCC). **Agglomération de Montréal**, Montréal, 9.VII.1975, C. Chantal, attracted to UV lamp (1, CCC). **MRC de Deux-Montagnes**, Oka, 4.VIII.2001, C. Chantal (1, CCC); Oka, parc national d’Oka, composting site, 45.4765N, 74.0542W, 26.VI.2015, R. Vigneault, flight interception trap (white tulle fabric) (1, RVC); Oka, rue Mont-Saint-Pierre, 13–27.IX.2017, R. Vigneault, attracted to UV lamp (2, RVC). **MRC de Marguerite-D’Youville**, Varennes, 15.VI.1998 (1), 22.VI.1998 (1), 15.VII.1998 (1), 4.VIII.2001 (1), 19.VII.2005 (1), 19.VIII.2009 (1), 31.VII.2010 (1), 3.VIII.2012 (1), C. Chantal, attracted to UV lamp (8, CCC). **MRC de Vaudreuil-Soulanges**, Terrasse-Vaudreuil, 45.3924N, 73.9922W, 20.V.2012, 14 h, P. de Tonnancour, decayed plant matter, compost heap (1, PdTC); same locality data and collector but 45.3923N, 73.9922W, 5.IX.2012, 23 h (12), 9.VII.2013, 22–24 h (1), 25.VIII.2014, 23 h (1), 31.VIII.2014, 21–24 h (2), 1.IX.2014, 21–24 h (3), 4.IX.2014, 20–24 h (2), 1.IX.2015, 20–24 h (2), 15.IX.2015, 23 h (1), 13.VII.2018, 22–23 h (1), 29.VIII.2018, 22 h (1), attracted to porch + UV lamps (26, PdTC); same locality data and collector but 13.VII.2013, 16 h, rotten banana peel, compost heap (1, PdTC); same locality data and collector but 16.VII.2018, 22 h, attracted to porch + UV + mercury vapor lamps (1, PdTC); same locality data and collector but 2627.VII.2018, 18–9 h (6), 9.X.2018, 18–19 h (5), window flight interception trap baited with ill-smelling plant compost (11, PdTC); same locality data and collector but 26.VIII.2018, 23 h, attracted to porch + mercury vapor lamps (9, PdTC).

**Distribution in Canada and Alaska.**BC, ON, QC (Klimaszewski et al. 2015, Pentinsaari et al. 2019).

#### Family Monotomidae Laporte, 1840

**Subfamily Rhizophaginae Redtenbacker, 1845**

***Rhizophagussayi* C. Schaeffer, 1913, new to Quebec**

**Note.** This species seems to be associated with deciduous trees, such as *Quercus* sp., American beech, American chestnut (*Castaneadentata* (Marsh.)), sugar maple (*Acersaccharum* Marsh.), and black cherry (*Prunusserotina* Ehrh.) (Bousquet, 1990).

**Specimen data. Quebec, MRC de Deux-Montagnes**, Oka, parc national d’Oka, ~1 km W of lac de la Sauvagine, 17.IV.2015, R. Vigneault (1, RVC); **MRC de Vau-dreuil-Soulanges**, Ville de l’Île-Perrot, 45.3958N, 73.9780W, 8.V.2019, 15 h (1), 20.V.2019, 16 h (1), P. de Tonnancour, large *Fagusgrandifolia* log (2, PdTC).

**Distribution in Canada and Alaska.**ON, **QC** (Bousquet et al. 2013).

#### Family Cryptophagidae Kirby, 1826

**Subfamily Cryptophaginae Kirby, 1826**

***Caenoscelisferruginea* (C.R. Sahlberg, 1820), new to New Brunswick**

**Specimen data. New Brunswick, Restigouche Co.**, ca. 3 km SE of Simpsons Field, 47.5377N, 66.5142W, 14–28.V.2015, C. Alderson & V. Webster // Old cedar & spruce forest with *Populustremuloides*, Lindgren funnel trap (1, RWC). **York Co.**, Canterbury, Eel River P.N.A., 45.8966N, 67.6345W, 6–21.V.2014, C. Alderson & V. Webster // Old-growth eastern white cedar swamp & fen, Lindgren funnel trap (1, RWC); Fredericton, Odell Park, 45.9508N, 66.6723W, 27.VI–5.VII.2017, C. Alderson & V. Webster // Old mixed forest, Lindgren funnel trap (1, RWC).

**Distribution in Canada and Alaska.**AK, YT, NT, BC, AB, SK, MB, ON, QC, **NB**, NS (Bousquet et al. 2013, Pelletier and Hébert 2019).

***Cryptophagusconfertus* Casey, 1900**

Bousquet et al. (2013) treated *C.confertus* as a synonym of *C.jakowlewi* Reitter, 1888. However, Esser (2018) showed that *C.jakowlewi* and *C.confertus* are distinct species and noted that there were no valid records of *C.jakowlewi* from North America. Webster et al. (2016c) newly reported *C.jakowlewi* from NB. These specimens were checked again using the keys in Woodroffe and Coombs (1961) and Pelletier and Hébert (2019) and they are *C.confertus*. Pelletier and Hébert (2019) did not list *C.confertus* for NB or note the original NB record of *C.jakowlewi* by Webster et al (2016c). This species is easily confused with the very similar and mostly western *C.bidentatus* Mäklin, 1853 that occurs as far east as QC (Pelletier and Hébert 2019).

***Henoticuspilifer* (Reitter, 1888) †, new to New Brunswick**

**Note.**Pelletier and Hébert (2019) newly reported the adventive Asian *Henoticuspilifer* from North America from QC, ON, and BC in Canada. Webster et al. (2012b) newly reported *H.serratus* (Gyllenhal, 1808) from NB. However, only one of these specimens (from McAdam, Georgia Pacific Plywood Mill, 19.V.1978 in the AFC collection) is *H.serratus*. The other specimens are *H.pilifer*, a new species for the province. Below we report the data from the Webster et al. (2012b) study and include a few more recent records of *H.pilifer*. Pelletier and Hébert (2019) show locality points for *H.serratus* in southeastern and northern NB indicating a broad distribution for this native Holarctic species. Interestingly, the adventive *H.pilifer* appears to be more common than *H.serratus* in southern NB.

**Specimen data. New Brunswick, Queens Co.**, Cranberry Lake P.N.A., 46.1125N, 65.6075W, 24.IV-V.2009, 5–12.V.2009, 12–21.V.2009, 21–27.V.2009, 27.V–5.VI.2009, 5–11.VI.2009, 11–18.VI.2009, 18–25.VI.2009, R. Webster & M.-A. Giguère, coll. // Mature red oak forest, Lindgren funnel traps (6, AFC; 2, NBM; 9, RWC); same locality data but 4–18.VIII.2011, M. Roy & V. Webster, coll. (1, RWC). **York Co**., Charters Settlement, 45.8395N, 66.7391W, 5.IX.2006, R.P. Webster // Mixed forest, among moldy corncobs and cornhusks (1, RWC); Fredericton, Odell Park, 45.9539N, 66.6666W, 10–24.VI.2012, C. Alderson & V. Webster // Hardwood forest, Lindgren funnel trap 1 m high under trees (1, AFC).

**Distribution in Canada and Alaska.**BC, ON, QC, **NB** (Pelletier and Hébert 2019).

**Subfamily Atomariinae LeConte, 1861**

***Atomariamorio* Kolenati, 1846 †, new to New Brunswick**

**Note.**Pelletier and Hébert (2019) reported this uncommon adventive European species for the first time from North America. Most NB specimens were captured in Lindgren funnel traps in various forest types. One individual was collected from the nest box contents of a barred owl (*Strixvaria* Barton). This species appears to be widespread in southern NB but was usually captured as singletons at a given locality.

**Specimen data. New Brunswick, Carleton Co.**, Jackson Falls, “Bell Forest”, 46.2200N, 67.7231W, 14–20.V.2009, R. Webster & M.-A. Giguère, coll. // Rich Appalachian hardwood forest with some conifers, Lindgren funnel trap (1, RWC). **Queens Co.**, Pleasant Villa, 45.7023N, 66.1732W, 15.VI.2007, S. Makepeace & R. Webster, coll. // Nest box contents of Barred Owl (6 litres), damp organic material with feathers & small bones (1, RWC); Jemseg, 45.8412N, 66.1195W, 28.V–12.VI.2012, C. Alderson, C. Hughes, & V. Webster // Hardwood woodland near seasonally flooded marsh, Lindgren funnel trap 1 m high under *Quercusmacrocarpa* (1, RWC). **Sunbury Co.**, Acadia Research Forest, 45.9866N, 66.3841W, 24–30.VI.2009, R. Webster & M.-A. Giguère, coll. // Red spruce forest with red maple & balsam fir, Lindgren funnel trap (1, RWC). **York Co.**, 15 km W of Tracy, off Rt. 645, 45.6848N, 66.8821W, 19–25.V.2009, R. Webster & M.-A. Giguère, coll. // Red pine forest, Lindgren funnel trap (1, RWC); Douglas, Currie Mountain, 45.9832N, 66.7564W, Old *Pinusstrobus* stand, Lindgren funnel trap, 1 m high under *P.strobus* (1, RWC); Keswick Ridge, 45.9962N, 66.8781W, 19.V–3.VII.2014 (1), 5–19.V.2015 (1), 3–18.VI.2015 (2), C. Alderson & V. Webster // Mixed forest, Lindgren funnel traps 1 m high under trees (4, RWC); Douglas, N.B. Walking Trail, 45.9819N, 66.7568W, 5–15.V.2015, C. Hughes & V. Webster // Hardwood forest, Lindgren funnel trap 1 m high under trees (1, RWC).

**Distribution in Canada and Alaska.**AB, SK, ON, QC, **NB**, NS (Pelletier and Hébert 2019).

***Atomariaimpressa* Erichson, 1846 †, new to New Brunswick**

**Note.**Pelletier and Hébert (2019) reported this adventive European species for the first time from North America, from strawberry fields and American beaver (*Castorcanadensis* Kuhl) lodges. The only specimen from NB with microhabitat data was collected from debris on an American beaver dam.

**Specimen data. New Brunswick, Carleton Co.**, Jackson Falls, “Bell Forest”, 46.2200N, 67.7231W, 9–14.V.2009, R. Webster & M.-A. Giguère, coll. // Rich Appalachian hardwood forest with some conifers, Lindgren funnel trap (1, AFC). **York Co.**, Charters Settlement, 45.8331N, 66.7279W, 20.V.2010, R.P. Webster, coll. // Beaver dam among sticks, debris, and clay on dam (1, RWC).

**Distribution in Canada and Alaska.**AB, ON, QC, **NB** (Pelletier and Hébert 2019).

***Atomariaplanulata* Mäklin, 1853, new to New Brunswick**

**Specimen data. New Brunswick, Gloucester Co.**, Bathurst, Daly Point Reserve, 47.6392N, 65.6098W, 15–25.VI.2015, C. Alderson & V. Webster // Mixed forest, black Lindgren funnel trap 1 m high (1, RWC). **Northumberland Co.**, ca. 1.5 km NW of Sevogle, 47.0939N, 65.8387W, 11–26.VI.2013 (2), 26.VI–8.VII.2013 (4), C. Alderson & V. Webster // *Populustremuloides* stand with a few conifers, Lindgren funnel traps in canopy of *P.tremuloides* (2, AFC; 1, NBM; 3, RWC). **Restigouche Co.**, Dionne Brook P.N.A., 47.9064N, 68.3441W, 31.V–15.VI.2011 (1), 15–27.VI.2011 (1), 27.VI–14.VII.2011 (1), M. Roy & V. Webster // Old-growth white spruce & balsam fir forest, Lindgren funnel traps (3, RWC); same locality and collectors but 47.9030N, 68.3503W, 27.VI–14.VII.2011 // Old-growth northern hardwood forest, Lindgren funnel traps (1, AFC; 2, NBM; 3, RWC); Jacquet River Gorge P.N.A., 47.8257N, 66.0764W, 10–25.VI.2014, Old *Populusbalsamifera* stand near river, Lindgren funnel trap 1 m high under trees (1, AFC; 2, NBM; 1, RWC). **York Co.**, Keswick Ridge, 45.9962N, 66.8781W, 3–18.VI.2015, C. Alderson & V. Webster // Lindgren funnel trap 1 m high under trees (1, NBM).

**Distribution in Canada and Alaska.**AK, YT, BC, AB, QC, **NB** (Bousquet et al. 2013, Pelletier and Hébert 2019).

#### Family Passandridae Blanchard, 1845

***Catogenusrufus* (Fabricius, 1792), new to New Brunswick**

**Note.** The *Catogenusrufus* specimen reported below represents the first record of the family Passandridae from NB.

**Specimen data. New Brunswick, York Co.**, Crabbe Mountain, 46.1208N, 67.1056W, 27.VII – 10.VIII.2018, C. Alderson & V. Webster // Hardwood forest, Lindgren funnel trap in tree canopy (1, RWC).

**Distribution in Canada and Alaska.**ON, QC, **NB** (Bousquet et al. 2013).

#### Family Phalacridae Leach, 1815

***Phalacruspolitus* Melsheimer, 1844, new supporting data for New Brunswick**

**Note.***Phalacruspolitus* was reported for the first time from Canada by Majka et al. (2008) based on specimens from two localities in NF. Bousquet et al. (2013) listed this species as occurring in NB. However, there is no published or supporting data in the CNC database for its occurrence in NB (Serge Laplante, pers. com.). Here, we report supporting data for NB.

All NB specimens of *P.politus* were collected from wetland habitats (marshes and fens, calcareous cedar fen, silver maple forest). Adults and larvae are associated with smuts on grasses (Steiner 1984).

**Specimen data. New Brunswick, Sunbury Co.**, Maugerville, Portobello Creek N.W.A., 45.8992N, 66.4248W, 5.VI.2004, R.P. Webster, coll. // Silver maple forest, sweeping foliage on margin of forest road (1, RWC). **York Co.**, Charters Settlement, 45.8282N, 66.7367W, 9.IV.2005, 16.IV.2015, R.P. Webster, coll. // *Carex* marsh, in leaf litter at base of trees & shrubs (2, RWC); same locality data and collector but 9.VI.2016 // Pond margin / marsh, sweeping vegetation (1, RWC); same locality and collector but 45.8267N, 66.7343W, 23.V.2005 // Sedge marsh & fen, treading saturated sphagnum/sedge hummocks into water (1, RWC); same locality data and collector but 8.VII.2005, 23.VII.2005 // Sedge marsh & fen, on flowers of *Spiraeaalba* (2, RWC); Canterbury, Eel River P.N.A., 45.8967N, 67.6343W, 20.VI.2014, R.P. Webster // Calcareous cedar fen with shrubby cinquefoil, sweeping vegetation with *Myricagale* & *Rhododendrongroenlandicum* (2, RWC); Spednic Lake P.N.A., near Pats Brook, 45.6210N, 67.4342W, 15.VI.2018, R.P. Webster // Freshwater marsh with slow flowing stream with emergent vegetation, sweeping vegetation (1, RWC).

**Distribution in Canada and Alaska.**ON, NB, NF (Bousquet et al. 2013).

#### Family Laemophloeidae Ganglbauer, 1899

***Charaphloeusconvexulus* (LeConte, 1879), new supporting data for Quebec**

**Note.** This species was added to the entomofauna of QC without any further details by Bousquet et al. (2013). Here, we provide supporting data for its occurrence in the province. Very little is known about its biology, except that it is usually found under bark. Most specimens listed below were captured in hardwood forests.

**Specimen data. Quebec, Agglomération de Montréal**, Dollard-des-Ormeaux, 30.V.1992. C. Chantal (1, CCC). **Agglomération de Québec**, Saint-Augustin, Portneuf, 26.VI.1982, C. Chantal (1, CCC); Saint-Étienne-de-Lauzon, Lévis, 9.V.1981 (1), 12.VI.1982 (1), C. Chantal (2, CCC). **MRC de Deux-Montagnes**, Oka, 14.VII.1982, C. Chantal (1, CCC); Oka, parc national d’Oka, 14.VI.1995, R. Vigneault (1, RVC); Oka, parc national d’Oka, La Grande Baie, 10.V.2003 (1), 13.V.2017, beating (1) (2, RVC); Oka, parc national d’Oka, Welcoming center, La Grande Baie, 15.VI.2003 (1, RVC); Oka, parc national d’Oka, wooded area ca. 1 km W of lac de la Sauvagine, 5.V.2019 (2), 19.V.2019 (1), 24.VI.2019 (1), R. Vigneault, flight interception trap (white tulle fabric) (4, RVC); Oka, parc national d’Oka, 4.VI.2016, 17 h, P. de Tonnancour, swept from understory vegetation (1, PdTC). **MRC de Joliette**, Joliette, 19.V.1979, C. Chantal, attracted to UV lamp (1, CCC). **MRC de Vaudreuil-Soulanges**, Notre-Dame-de-l’Île-Perrot, 14.V.2016, 15 h, P. de Tonnancour, underside of freshly cut *Acersaccharum* log (1, PdTC); Ville de l’Île-Perrot, 45.3958N, 73.9780W, 15.V.2019, 16 h (1), 16.V.2019, 14 h (5), 20.V.2019, 16 h (2), 25.V.2019 (1), 30.V.2019 (1), P. de Tonnancour, large *Fagusgrandifolia* log (10, PdTC).

**Distribution in Canada and Alaska.**ON, QC, NB, NS (Bousquet et al. 2013).

***Lathropusvernalis* Casey, 1884, new supporting data for Quebec**

**Note.** The identity of the first specimen recorded below was confirmed by Michael C. Thomas in 2017.

**Specimen data. Quebec, Agglomération de Longueuil**, Boucherville, 3.VIII.1992, C. Chantal (1, CCC); Longueuil, 25.VII.1992, C. Chantal // Beating (1, CCC). **Agglomération de Montréal**, Montréal, parc Zotique-Racicot, 45.541392N, 73.682889W, 28.VI.2016, 13 h, P. de Tonnancour, beaten from *Ulmusamericana* (1, PdTC). **MRC de Marguerite-d’Youville**, 6.VII.2000, C. Chantal // *In copula* (2, CCC). **MRC de Vaudreuil-Soulanges**, Saint-Lazare, (45.3807N, 74.1691W), 28.VII.2016, 14 h, P. de Tonnancour, beaten from dead *Pinussylvestris* (with needles still attached) (1, PdTC).

**Distribution in Canada and Alaska.**SK, QC (Bousquet et al, 2013)

***Placonotusfalinorum* Thomas, 2011, new to Canada and Quebec**

**Note.** The identity of one of the two specimens reported below was confirmed by Michael C. Thomas in 2016 (see M.C. Thomas comments and photo of the specimen at https://bugguide.net/node/view/1100475/bgpage).

**Specimen data. Quebec, MRC de Deux-Montagnes**, Oka, parc national d’Oka, Calvaire d’Oka, 30.IV.2001, R. Vigneault (1, RVC); Oka, parc national d’Oka, La Grande Baie, 5.VII.2015, R. Vigneault, beaten from dead branches, deciduous forest (1, RVC).

**Distribution in Canada and Alaska. QC**.

#### Family Nitidulidae Latreille, 1802

**Subfamily Epuraeinae Kirejtshuk, 1986**

***Epuraeafulvescens* Horn, 1879, new to New Brunswick**

**Specimen data. New Brunswick, Carleton Co.**, Belleville, Meduxnekeag Valley Nature Preserve, 46.1888N, 67.6762W, 13.VIII.2007, R.P. Webster, coll. // River margin, sweeping flowers of *Daucuscarota* (3, RWC); Jackson Falls, “Bell Forest”, 46.2200N, 67.7231W, 8.VII.2004, R.P. Webster, coll. // Rich Appalachian hardwood forest, m.v. light (5, RWC). **Queens Co.**, Cranberry Lake PNA, 46.1125N, 65.6075W, 5–11.VI.2009, R. Webster & M.-A. Giguère // Red oak forest, Lindgren funnel trap (1, RWC). **York Co.**, Charters Settlement, 45.8395N, 66.7391W, 16.VI.2007, R.P. Webster, coll. // Mixed forest, on flowers of ornamental *Spiraea* species (1, RWC).

**Distribution in Canada and Alaska.**ON, QC, **NB** (Bousquet et al. 2013).

**Subfamily Carpophilinae Erichson, 1842**

***Carpophilusmelanopterus* Erichson, 1843, new supporting data for Quebec**

**Note.** This species is closely associated with *Yucca* spp. both in the larval and adult stages. Parsons (1943) recorded it as ranging in the United States from NY, NJ, and IL south to FL and west to IA and TX. The CNC contains specimens from ON (Windsor, 3.VIII.1954, ex. *Yuccafilamentosa* L. [6 specimens]; Ancaster, 7.VII.1978, on *Yucca* flowers; Ottawa, 5.VII.2012, yucca, H. Goulet [4 specimens]), but since no potential host *Yucca* sp. grows native in the province, McNamara in Bousquet et al. (1991) considered the Windsor occurrence doubtful or incidental and opted not to include this species in the checklist. The recent discovery of a few specimens in Ottawa by H. Goulet and of the specimens recorded hereafter, which were the basis of the record from QC reported by Bousquet et al. (2013), confirms its presence in both ON and QC. The popularity of yuccas as garden plants in southern Canada has likely played a key role in the northward range expansion of this species. Label data suggest that the adults are mostly active in late afternoon and early evening during the first half of July, when their host plants are in bloom.

**Specimen data. Quebec, Agglomération de Longueuil**, Boucherville, 7.VII.2011, 19 h (3), 10.VII.2011, 19 h (3), G. Lafrance & L. de Tonnancour, flower stalk of *Yuccafilamentosa* (2, CTC; 4, PdTC). **MRC de Marguerite-D’Youville**, Varennes, 7.VII.2012 (1), 4.VII.2013 (3), 30.VI.2014 (5), C. Chantal, *Yucca* sp. (9, CCC). **MRC de Vaudreuil-Soulanges**, Terrasse-Vaudreuil, 45.3923N, 73.9925W, 1.VII.2011, 18 h (5), 4.VII.2011, 20 h (16), 7.VII.2011, 19 h (3), 10.VII.2011, 19 h (1), 10.VII.2015 (6), 4.VII.2016, 12 h (1), 9.VII.2019, 12 h (2), P. de Tonnancour, flower stalk of *Yuccafilamentosa* (6, SDC; 26, PdTC); same locality and biological data but 18.VII.2017, S. Dumont (1, SDC).

**Distribution in Canada and Alaska.**ON, QC (Bousquet et al. 2013).

**Subfamily Amphicrossinae Kirejtshuk, 1986**

***Amphicrossusciliatus* (Olivier, 1811), new supporting data for Quebec**

**Note.** Label data presented below indicate that *Amphicrossusciliatus* has been present in QC since at least 1993. Parsons (1943) mentioned that this species is found at sap in the spring but occurs on flowers of *Eupatorium* and allied plants in the autumn. However, the vast majority of the specimens reported below have been collected at light.

**Specimen data. Quebec, Agglomération de Montréal**, Sainte-Anne-de-Bellevue, 12.VI.1993, C. Chantal (1, CCC); **MRC de Deux-Montagnes**, Oka, parc national d’Oka, 26.VI.2016, 23 h, P. de Tonnancour, wooded area next to beach, attracted to mercury vapor lamp (1, PdTC); **MRC de Laval**, Laval, 30.VI.1997, R. Vigneault (2, RVC); **MRC Des-Jardins-de-Napierville**, Saint-Bernard-de-Lacolle, 16.VII.1994, C. Chantal (1, CCC); **MRC de la Vallée-du-Richelieu**, Mont-Saint-Hilaire, 45.5364N, 73.1590W, 23.VI.2013, Henri Miquet-Sage (1, PdTC); **MRC de Marguerite-D’Youville**, Varennes, 16.VII.1997 (1), 11.VI.1998 (1), 22.VI.1998 (1), 23.VI.1998 (1), 29.VI.1998 (1), 6.VI.1999 (1), 3.VII.1999 (1), 2.VI.1999 (1), C. Chantal, attracted to UV lamp (8, CCC); **MRC de Vaudreuil-Soulanges**, Terrasse-Vaudreuil, 1.VII.2007, 23 h (1), 12.VII.2007, 22 h (1), 31.VII.2010, 23 h (1), P. de Tonnancour, attracted to UV lamp (3, PdTC); same locality data and collector, but 30.V.2011, 22 h (2), 18.VII.2011, 0 h (1), 13.VIII.2011, 23 h (1), 28.VI.2012, 23 h (1), 1.IX.2012, 1 h (1), 30.V.2013, 21 h (1), 15.VII.2013, 23 h (1), 25.VIII.2013, 21 h (1), 28.V.2014, 22 h (2), 25.VI.2014, 23 h (1), 6.IX.2014, 23 h (1), 28/29.V.2016, 23–1 h (2), 1.VI.2016, 22 h–23 h 30 (2), 2.VI.2016, 22–24 h (3), 3.VI.2016, 22–23 h (2), 18.VI.2016, 23 h (2), 15.VII.2016, 23 h (1), 17.VIII.2016, 22 h (1), attracted to porch + UV lamps (26, PdTC); Mont Rigaud, 45.4636N, 74.2711W, 4.VII.2013, 23 h, P. de Tonnancour, attracted to UV lamp (1, PdTC); **MRC du Haut-Saint-Laurent**, Dundee, 23.V.2010, attracted to UV lamp, C. Tessier, *Juglansnigra* stand (1, CTC).

**Distribution in Canada and Alaska.**ON, QC (Bousquet et al. 2013).

**Subfamily Nitidulinae Latreille, 1802**

***Stelidotageminata* (Say, 1825), new supporting data for Quebec**

**Note.** The 2011 specimens reported below were the basis of the record from QC reported by Bousquet et al. (2013).

**Specimen data. Quebec, MRC de Marguerite-D’Youville**, Varennes, 20.VI.2012 (2), 29.VI.2012 (1), 22.VII.2012 (1), 26.VI.2016 (1), 4.VII.2016 (1), C. Chantal, attracted to UV lamp (6, CCC). **MRC de Brome-Missisquoi**, Saint-Armand, 5.VII.2016, C. Chantal, attracted to UV lamp (1, CCC). **MRC de D’Autray**, Berthierville, 9.VI.2009, C. Chantal, attracted to UV lamp (1, CCC). **MRC de Vaudreuil-Soulanges**, Terrasse-Vaudreuil, 45.3924N, 73.9921W, 26.IX.2011, 14 h, P. de Tonnancour, fermented cantaloupe (9, PdTC); Terrasse-Vaudreuil, 45.3875N, 73.9906W, 5.VI.2011, 16 h, P. de Tonnancour, swept from *Erysimumcheiranthoides* (1, PdTC); Terrasse-Vaudreuil, 45.3923N, 73.9922W, 9.V.2013, 18 h (1), 31.V.2013, 19 h (2), 25.VIII.2013, 18 h (1), 15.IX.2013, 18 h (7), P. de Tonnancour, flight interception trap (white tulle fabric) (11, PdTC); 45.3924N, 73.9921W, 20.VI.2013, 17 h, P. de Tonnancour, fermented pineapple (10, PdTC); Terrasse-Vaudreuil, 26–27.VII.2018, 18–9 h, P. de Tonnancour, window flight interception trap baited with ill-smelling plant decayed matter (1, PdTC).

**Distribution in Canada and Alaska.**ON, QC (Bousquet et al. 2013).

#### Family Anamorphidae Strohecker, 1953

***Symbiotesduryi* Blatchley, 1910, new to Quebec**

**Note.** This native species was known to occur in Canada exclusively in ON (Bousquet et al. 2013) until Webster et al. (2016) recorded its presence in NB.

**Specimen data. Quebec, MRC de Deux-Montagnes**, Oka, parc national d’Oka, 45.4744N, 74.0362W, 9.VII.2019, 16–19 h 30 (1), 19.VII.2019, 17 h 30–19 h (1), flight interception trap (white tulle fabric), red oak-white pine stand, P. de Tonnancour (2, PdTC).

**Distribution in Canada and Alaska.**ON, **QC**, NB (Webster et al. 2016).

#### Family Coccinellidae Latreille, 1807

Majka and McCorquodale (2010) reported 40 coccinellid species from NB. Five additional species were added to the NB faunal list by Webster et al. (2012d, 2016c) and 45 species were recognized as established in NB by Webster (2016c). Most recently, McAlpine et al. (2018) reported *Coleomegillamaculatalengi* Timberlake, 1943 from the province. Members of the genus *Scymnus* can be difficult to determine due to variation in coloration and size, and many species can only be determined with certainty by dissecting males (Gordon 1976). A review of *Scymnus* and *Stethorus* specimens in several NB collections resulted in the discovery of seven additional species of *Scymnus* and one adventive *Stethorus* species, bringing the total number of NB coccinellids to 54 species. These new records are reported below.

#### Subfamily Coccinellinae Latreille, 1807

***Scymnusabbreviatus* LeConte, 1852, new to New Brunswick**

**Note.**Gordon (1976) reported *Scymnusabbreviatus* from the Great Lakes area and along the St. Lawrence River in ON, QC, and MI and suggested it should be found throughout the region north of the Great Lakes. Majka et al. (2011) did not report it from ME. Its presence in NB is somewhat surprising.

Specimens of *S.abbreviatus* were collected by sweeping vegetation along a roadside and in a brushy area. One was collected from trembling aspen and three were captured in Lindgren funnel traps (that were either all green or all black) in a mixed forest. Adults were collected during May and June. No habitat data for this species were provided by Gordon (1976).

**Specimen data. New Brunswick, Gloucester Co.**, Bathurst, Daly Point Reserve, 47.6392N, 65.6098W, 13–28.V.2015, C. Alderson & V. Webster // Mixed forest, green Lindgren funnel trap 1 m high (1 ♂ (dissected), RWC); same data as previous but black Lindgren funnel traps (2 ♀♀, RWC). **Queens Co.**, 2 mi W of S. Minto, 4.VI.1962 // 62-0138-01, ex. trembling aspen // 640811 // AFCF0010387 (1 ♂ (dissected), AFC). **Sunbury Co.**, Maugerville, off Rt. 105, 45.8662N, 66.4559W, 4.VI.2013, R.P. Webster // Flood plain forest, sweeping roadside vegetation (1 ♂ (dissected), RWC). **York Co.**, New Maryland, 45.8430N, 66.7275W, 17.VI.2007, R.P. Webster, coll. // Regenerating mixed forest, sweeping foliage in brushy area (1 ♀, RWC).

**Distribution in Canada and Alaska.**ON, QC, **NB** (Bousquet et al. 2013).

***Scymnuscaudalis* LeConte, 1850, new to New Brunswick**

**Note.**Majka and McCorquodale (2006) recorded this species from Halifax, NS as a “seemingly isolated” population. This species is common and broadly distributed in eastern and central North America (Gordon 1976) and appears to be widespread in NB.

Two individuals were captured in Lindgren funnel traps in a hardwood forest and a mixed forest. The only specimen with specific habitat data was swept from foliage along a lakeshore on an old dune with oaks. No habitat data for this species were provided by Gordon (1976) or Majka and McCorquodale (2006, 2010).

**Specimen data. New Brunswick, Carleton Co.**, Jackson Falls, “Bell Forest”, 46.2200N, 67.7231W, 12–19.VI.2008, R.P. Webster, coll. // Rich Appalachian hardwood forest with some conifers, Lindgren funnel trap // AFCF0010382 (1 ♂ (dissected), AFC). **Gloucester Co.**, Bathurst, Daly Point Reserve, 47.6392N, 65.6098W, 28.V–15.VI.2015, C. Alderson & V. Webster // Mixed forest, green Lindgren funnel trap 1 m high (1 ♂ (dissected), RWC). **Queens Co.**, Canning, Grand Lake near Scotchtown, 45.8762N, 66.1816W, 1.VII.2004, D. Sabine & R. Webster, coll. // Lakeshore, old dune with oaks, sweeping foliage (1 ♂ (dissected), RWC).

**Distribution in Canada and Alaska.**SK, MB, ON, QC, **NB**, NS (Bousquet et al. 2013).

***Scymnuspuncticollis* LeConte, 1852, new to New Brunswick**

**Note.**NB specimens were captured in Lindgren funnel traps in a hardwood forest, a mixed forest with red oak, a mixed forest, and an old balsam poplar (*Populusbalsamifera* L.) stand near a river. Most of these were captured in traps in the forest canopy. Adults with specific habitat data were collected by beating or sweeping foliage in a regenerating mixed forest and in a sand pit. Adults were collected from April to September. No habitat data were provided by Gordon (1976). This appears to be the most common and widespread *Scymnus* species in NB.

**Specimen data. New Brunswick, Carleton Co.**, Jackson Falls, “Bell Forest”, 46.2200N, 67.7231W, 13–25.IV.2012, R. Webster, J. Sweeney, & C. Hughes // Rich Appalachian hardwood forest with some conifers, Lindgren funnel trap in canopy of *Acersaccharum* // AFCF0018637 (1 ♂ (dissected), AFC). **Queens Co.**, C.F.B. Gagetown, 45.7516N, 66.1866W, 9–22.V.2013, C. Alderson & V. Webster // Old mixed forest with *Quercusrubra*, Lindgren funnel trap in canopy of *Q.rubra* // AFCF0018639 (1 ♂ (dissected), AFC); same data but 22.V–4.VI.2013 // AFCF0018640 (1 ♂ (dissected), AFC). **Restigouche Co.**, Jacquet River Gorge P.N.A., 47.8257N, 66.0764W, 10–25.VI.2014, C. Alderson & V. Webster // Old *Populusbalsamifera* stand near river, Lindgren funnel trap 1 m high under trees (1 ♂ (dissected), RWC). **York Co.**, New Maryland, 45.8428N, 66.7279W, 5.VI.2003, R.P. Webster, coll. // Regenerating mixed forest, beating foliage (1 ♂ (dissected), RWC); New Maryland, Charters Settlement, 45.8430N, 66.7275W, 17.VI.2004, R.P. Webster, coll. // Regenerating mixed forest, sweeping foliage (1 ♂ (dissected), NBM, 1 ♂ (dissected), RWC); same data as previous but 12.VII.2005 // Regenerating mixed forest, beating foliage (1 ♂ (dissected), NBM): Upper Brockway near abandoned airport, 45.5729N, 67.0959W, 28.V.2018, R.P. Webster // Sand pit, beating *Salix* foliage (1 ♂ (dissected), RWC); Spednic Lake P.N.A., 45.6751N, 67.4726W, 10–24.V.2018 (1), 31.VII–16.VIII. 2018 (2), 16–30.VIII.2018 (3), 30.VIII–12.IX.2018 (1), C. Alderson & V. Webster // Mixed forest, Lindgren funnel traps in tree canopies (1 ♂ (dissected), AFC; 2 ♂♂ (dissected), NBM; 4 ♂♂ (dissected) , RWC); Crabbe Mountain, 46.1208N, 67.1056W, 13–27.VII.2018 (1), 27.VII – 10.VIII.2018 (1), 24.VIII – 6 IX.2018 (1), C. Alderson & V. Webster // Hardwood forest, Lindgren funnel traps in tree canopies (1 ♂ (dissected), AFC; 2 ♂♂ (dissected), RWC).

**Distribution in Canada and Alaska.**ON, QC, **NB** (Bousquet et al. 2013).

***Scymnussecurus* J. Chapin, 1973, new to New Brunswick**

**Note.***Scymnussecurus* is a coastal plain species recorded from LA north to MA with an isolated record from Tilbury, ON (Gordon, 1976, 1985). The record from NB represents a significant range extension to the northeast. The three known NB specimens were sifted from moist grass litter in a small sedge marsh and swept from marsh vegetation along the margin of a beaver pond. Adults were collected in April, July, and September. No habitat data were provided by Gordon (1976).

**Specimen data. New Brunswick, York Co.**, Charters Settlement, 45.8428N, 66.7279W, 28.IV.2004, R.P. Webster, coll. // Mixed forest, small sedge marsh, in moist grass litter (1 ♀, RWC); same data as previous but 11.VII.2005 (1 ♀, NBM); Charters Settlement, 45.8296N, 66.7347W, 20.IX.2017, R.P. Webster // Beaver pond margin, sweeping vegetation (1 ♂ (dissected), RWC).

**Distribution in Canada and Alaska.**ON, **NB** (Bousquet et al. 2013).

***Scymnussuturalis* Thunberg, 1795 †, new to New Brunswick**

**Note.***Scymnussuturalis* appears to be widespread in NB but is apparently uncommon as only three specimens have been collected. This adventive Palaearctic species was first reported from Canada from Oka, QC in 1983 (McNamara 1992) and from Halifax, NS in 1993 by Hoebeke and Wheeler (1996) and was still present in Halifax in 2003 (Majka and McCorquodale 2010). The introduction into NS was likely an accidental one, possibly associated with conifer nursery stock (Hoebeke and Wheeler 1996, Majka and Klimaszewski 2004). This species was intentionally released in MI from Germany in 1961 and is apparently now established in the state and was also inadvertently introduced to other areas in the Northeast where it has become established (Gordon 1985).

*Scymnussuturalis* preys on adelgids and aphids found on conifers and has been found on Scots pine (*Pinussylvestris* L.) and jack pine in NS (Majka and McCorquodale 2010). The three NB specimens reported below were captured in Lindgren funnel traps; one in an old black spruce (*Piceamariana* (Mill.) BSP) forest, one in a red oak (*Quercusrubra* L.) forest near a seasonally flooded marsh, and another in a mixed forest. The specimens were captured from June to September.

**Specimen data. New Brunswick, Northumberland Co.**, Upper Graham Plains, 47.1001N, 66.8154W, 21.VIII–4.IX.2014, C. Alderson & V. Webster // Old black spruce forest, Lindgren funnel trap (1, RWC). **Sunbury Co.**, Sunpoke Lake, 45.7656N, 66.5550W, 9–20.VII.2012, C. Alderson & V. Webster // Red oak forest near seasonally flooded marsh, Lindgren funnel trap 1 m high under *Quercusrubra* (1, RWC). **York Co.**, Keswick Ridge, 45.9962N, 66.8781W, 4–16.VI.2014, C. Alderson & V. Webster // Mixed forest, Lindgren funnel trap in canopy (1, RWC).

**Distribution in Canada and Alaska.**QC, **NB**, NS (Bousquet et al. 2013).

***Scymnustenebrosus* Mulsant, 1850, new to New Brunswick**

**Specimen data. New Brunswick, Gloucester Co.**, Bathurst, Daly Point Reserve, 47.6392N, 65.6098W, 13–28.V.2015, C. Alderson & V. Webster // Mixed forest, black Lindgren funnel trap 1 m high (1 ♂ (dissected), RWC).

**Distribution in Canada and Alaska.**NT, AB, SK, MB, ON, QC, **NB**, NS, PE (Bousquet et al. 2013).

***Scymnusamericanus* Mulsant, 1850, new to New Brunswick**

**Note.***Scymnusamericanus* is widespread in the central and eastern USA and was reported close to the NB border in eastern ME (Gordon 1976). The single specimen from NB was sifted from moist grass litter in a small sedge marsh in April. No habitat data were provided by Gordon (1976).

**Specimen data. New Brunswick, York Co.**, Charters Settlement, 45.8428N, 66.7279W, 15.IV.2005, R.P. Webster, coll. // Mixed forest, small sedge marsh, in moist grass litter (1 ♀, RWC).

**Distribution in Canada and Alaska.**ON, QC, **NB** (Bousquet et al. 2013).

***Stethoruspunctillum* Weise, 1891 †, new to New Brunswick**

**Note.** The earliest record of this Palaearctic species from North America is from Leamington, ON in 1931 with later records from Lulu Island, BC in 1950 and Saint-Nicolas (Lévis), QC in 1993 (Brown, 1950, Klimaszewski et. 2015). It was first reported from the United States from Framingham, MA sometime before 1950 (Brown 1950). This species was inadvertently introduced and appears to have now become established throughout much of the Northeast west to MB, AB, southward to ID and on the west coast in BC and OR (Gordon, 1985, Klimaszewski et al. 2015). Majka et al. (2011) reported it from ME and thus its presence in NB is not unexpected. This species is easily confused with the native *S.punctum* (LeConte, 1852) and has been confused in collections (Gordon 1985). Re-examination of specimens previously determined as *S.punctum* may result in the discovery of additional localities for *S.punctillum* in the region.

*Stethoruspunctillum* feed on mites and soft-bodied insects such as aphids, adelgids, and scales (Gordon 1985, Klimaszewski et al. 2015). The NB specimens were captured in Lindgren funnel traps in an old jack pine forest, an old red oak forest, and a hardwood woodland near a seasonally flooded marsh. Adults were collected from late May to late August.

**Specimen data. New Brunswick, Northumberland Co.**, ca. 2.5 km W of Sevogle, 47.0879N, 65.8585W, 27.V–11.VI.2014, C. Alderson & V. Webster // Old *Pinusbanksiana* forest, Lindgren funnel trap (1, RWC). **Queens Co.**, Cranberry Lake P.N.A., 46.1125N, 65.6075W, 25.V–7.VI.2011, M. Roy & V. Webster, coll. // Old red oak forest, Lindgren funnel trap (1, RWC); Jemseg, 45.8412N, 66.1195W, 8–21.VIII.2012, C. Alderson & V. Webster // Hardwood woodland near seasonally flooded marsh, Lindgren funnel trap 1 m high under *Quercusmacrocarpa* (1, RWC).

**Distribution in Canada and Alaska.**BC, AB, SK, MB, ON, QC, **NB** (Bousquet et al. 2013, Klimaszewski et al. 2015).

#### Family Latridiidae Erichson, 1842

**Subfamily Latridiinae Erichson, 1842**

***Dienerellacostulata* (Reitter, 1877) †, new to New Brunswick**

**Specimen data. New Brunswick, Westmorland Co.**, Sackville, Morgan Lane, 45.9001N, 64.3651W, house, basement, J. Klymko (1, RWC; 3 NBM).

**Distribution in Canada and Alaska.**SK, MB, ON, QC, **NB**, NS, PE (Bousquet et al. 2013).

#### Family Mordellidae Latreille, 1802

**Subfamily Mordellinae Latreille, 1802**

***Falsomordellistenahebraica* (LeConte, 1862), new to New Brunswick**

**Specimen data. New Brunswick, Gloucester Co.**, Bathurst, Daly Point Preserve, 47.6392N, 65.6098W, 5–21.VIII.2015, C. Alderson & V. Webster // Mixed forest, green Lindgren funnel trap 1 m high (1, RWC).

**Distribution in Canada and Alaska.**MB, ON, QC, **NB** (Bousquet et al. 2013).

***Mordellinaandreae* LeConte, 1862, new to New Brunswick**

**Note.**Bousquet et al (2013) placed this species in the genus *Mordellina*. However, Lisberg (2003) recommended keeping this species in the genus *Mordellistena* but noted that it was not well placed in either genus. A specimen (not available) of this species from Edmundston, Madawaska Co. was photographed by Richard Migneault on June 12, 2017. See https://bugguide.net/node/view/1481467.

**Specimen data. New Brunswick, York Co.**, Charters Settlement, 45.8430N, 66.7275W, 27.VI.2004, R.P. Webster, coll. // Regenerating mixed forest, sweeping foliage (1, RWC); Spednic Lake PNA, nr. Palfrey Stream at East Brook Rd., 45.6984N, 67.4968W, 16.VIII.2017, R.P. Webster // Slow flowing stream with emergent vegetation, sweeping foliage (1, RWC).

**Distribution in Canada and Alaska.**ON, QC, **NB** (Bousquet et al. 2013).

***Mordellistenadivisa* LeConte, 1859, new to New Brunswick**

**Note.** It was surprising to find this distinctive species in NB. The NB specimens clearly key out to *Mordellistenadivisa* and fit the description in Liljeblad (1945) and appear identical to the type specimen in the MCZ Type Database @ Harvard Entomology. This species has been reported from NJ and NY in the eastern United States but appears to be mainly mid-western in distribution (Downie & Arnett 1996). Adults were swept from vegetation in an old field with open bare sandy areas.

**Specimen data. New Brunswick, Sunbury Co.**, 9.5 km NE Jct. 101 & 645, 45.7586N, 66.6755W, 17.VII.2008, R.P. Webster, coll. // Old field with open sandy areas, sweeping foliage (10, RWC).

**Distribution in Canada and Alaska.**SK, MB, **NB** (Bousquet et al. 2013).

***Mordellistenasericans* Fall, 1907, new to New Brunswick**

**Specimen data. New Brunswick, Sunbury Co.**, Sunpoke Lake, 45.7656N, 66.5550W, 5–15.VIII.2012, 5–27.VIII.2012, C. Alderson & V. Webster // Red oak forest near seasonally flooded marsh, Lindgren funnel trap 1 m high under *Quercusrubra* (2, RWC).

**Distribution in Canada and Alaska.**SK, MB, **NB**, NS, PE (Bousquet et al. 2013).

#### Family Meloidae Gyllenhal, 1810

**Subfamily Nemognathinae Laporte, 1840**

***Tricraniasanguinipennis* (Say, 1823)**

**Note.***Tricraniasanguinipennis* was first reported from NB on BugGuide.Net, https://bugguide.net/node/view/1512612/bgimage by Anthony W. Thomas, based on a photograph of an individual taken on 24 April, 2018 in Fredericton (Douglas). The individual was not collected (A. Thomas, pers. comm., 2019). This is the first record of this species from the province. The first author visited the same site on 5 May 2019 and collected four individuals representing the first specimen vouchers. Two additional individuals were observed on 6 May at the same site. Adults were observed crawling on the ground in areas of short grass with bare patches of sandy soil in or near colonies of ground nesting bees. Larvae of this genus are parasitoids of ground nesting bees and are phoretic on adult bees. Phoresy appears to be the main means this flightless species uses to reach their host nests (Pinto and Bologna 2002) and may also be the primary way it disperses to new areas.

This meloid has been reported as far north as Clinton, Kennebec Co., in central ME (Majka et al. 2011) and is known from QC and ON in Canada (Bousquet et al. 2013). The population in NB represents a significant range extension to the northeast. This species is currently known from only one locality in NB but may be more widespread. Adults may have been overlooked because they are active very early in the spring when red maples (*Acerrubrum* L.) are in flower.

**Specimen data. New Brunswick, York Co.**, Douglas, 45.9779N, 66.6859W, 5.V.2019, R.P. Webster // Area with short grass and sandy soil, on ground in area with ground nesting bees (2, NBM; 2, RWC).

**Distribution in Canada and Alaska.**ON, QC, **NB** (Bousquet et al. 2013).

#### Family Tenebrionidae Latreille, 1802

**Subfamily Diaperinae Latreille, 1802**

***Adelina pallida* (Say, 1824), new to Canada and Quebec**

**Note.***Adelina pallida* was known to occur in the United States as far north as IN, OH, and MD (Bousquet et al. 2018). The record from QC represents a significant northward range extension and the first occurrence of this species and of its genus in Canada. This small species is readily recognized among other members of its family by its markedly flat cucujid-like body.

**Specimen data. Quebec, MRC de Deux-Montagnes**, Oka, parc national d’Oka, 27.VII.2006, wooded area next to beach, R. Vigneault (1, RVC).

**Distribution in Canada and Alaska. QC**.

#### Family Anthicidae Latreille, 1819

**Subfamily Anthicinae Latreille, 1819**

***Sapintusfulvipes* (Laferté-Sénectère, 1847)**

This species was reported for the first time from NB on BugGuide.Net by Richard Migneault. See https://bugguide.net/node/view/861873 for a photograph of the specimen. The specimen, determined by D.S. Chandler, was collected in Edmundston, NB (Madawaska Co.) on June 6, 2013.

**Distribution in Canada and Alaska.**BC, SK, MB, ON, QC, **NB**, NS (Bousquet et al. 2013).

#### Family Cerambycidae Latreille, 1802

**Subfamily Lamiinae Latreille, 1825**

***Acanthocinusobsoletus* (Olivier, 1795), new to Nova Scotia**

**Specimen data. Nova Scotia, Annapolis Co.**, Kejimkujik N.P., 44.39889N, 65.21999W, 30.VII–13.VIII.2018, G. Marten-Carpenter, coll. // Mixed forest, Lindgren funnel trap, Trap 4 (1, AFC). **Queens Co.**, Kejimkujik National Park, 44.30972N, 65.32986W, 30.VII–17.VIII.2018, G. Marten-Carpenter, coll. // Mixed forest, Lindgren funnel trap, Trap 12 (9, AFC).

**Distribution in Canada and Alaska.**ON, QC, **NS** (Bousquet et al. 2017).

***Eupogoniuspauper* LeConte, 1852, new to New Brunswick**

**Note.** This longhorn has been reported as far north as the Quebec City area of QC and uses a wide variety of deciduous trees, shrubs and vines as host plants (Bousquet et al. 2017). Its presence in NB is not unexpected.

**Specimen data. New Brunswick, York Co.** Kingsclear, 45.9458N, 66.7948W, 8–21.VII.2017, C. Alderson & V. Webster // Mixed forest, Green 5-Funnel Lindgren funnel trap in tree canopy (1, AFC).

**Distribution in Canada and Alaska.**MB, ON, QC, **NB** (Bousquet et al. 2017).

***Mecascineracea* Casey, 1913, new to Manitoba**

**Note.** The first specimen of *Mecascineracea* reported from Canada was swept from vegetation near Willow Bunch Lake, SK in 1991 (Bousquet et al. 2017). The new MB record represents the second known Canadian specimen; it was swept from vegetation in a marshy area in a native tall grass prairie.

**Specimen data. Manitoba**, near Jct. 36N, 144W, N of Grande Clairière, 49.5289N, 100.7324W, 8.VII.2007, R.P. Webster, coll. // Native tall grass prairie, sweeping vegetation in marshy area (1, RWC).

**Distribution in Canada and Alaska.**SK, **MB** (Bousquet et al. 2017).

***Tetropspraeusta*** (**Linnaeus, 1758) †, new to Nova Scotia**

**Note.** This adventive species is common and widespread in NB (Webster and Sweeney, unpublished data). Its presence in NS is not unexpected.

**Specimen data. Nova Scotia, Halifax Co.**, Magazine Hill, 44.7143N, 63.6331W, 17.VI.2018, K. Van Rooyen & J. Palmer // Hardwood dominated forest, green Lindgren funnel trap in tree canopy (1, AFC)

**Distribution in Canada and Alaska.**ON, QC, NB, **NS** (Bousquet et al. 2013, 2017).

#### Family Chrysomelidae Latreille, 1802

**Subfamily Bruchinae Latreille, 1802**

***Acanthoscelidestenuis* Bottimer, 1935, new to New Brunswick and Quebec**

**Note.** As Kingsolver (2004) noted, “The genus *Acanthoscelides* is a large and diverse, poorly defined aggregate of mostly small species of Bruchidae”. Identification often requires an examination of the genitalia. The identification of the specimens from QC and NB is the result of a joint effort by H. Douglas and K. Savard (CNC), Geoffery Morse (U. of San Diego), and G.J. Kergoat (INRAE, France). *Acanthoscelidestenuis* was known to occur in ON, but the records presented here represents a significant range extension to the northeast.

**Specimen data. New Brunswick**, **Carleton Co.**, Jackson Falls, 46.2251N, 67.7401W, 24.VIII.2017, R.P. Webster // River margin, sweeping *Lythrumsalicaria* (1, RWC). **Quebec, MRC de Deux-Montagnes**, Oka, parc national d’Oka, 14.VI.2017, R. Vigneault, beating in a field at the western end of the park (2, RVC). **MRC Vaudreuil-Soulanges**, Notre-Dame-de-l’Île-Perrot, 8.VI.2011, 13 h, P. de Tonnancour, swept from *Barbareavulgaris* (1, PdT); same locality data and collector but 19.VI.2011, 13 h, beaten from *Cornusobliqua* inflorescence (2, CNC; 5, PdTC); same locality data and collector but 19.VII.2011, 15 h, beaten from *Scirpus* sp. (1, PdTC); same locality and collector but 12.VIII.2011, 15 h, swept from flowering *Ambrosiaartemisiifolia* (1, PdTC); Notre-Dame-de-l’Île-Perrot, 45.3757N, 73.9457W, 1.VIII.2018, 14 h, P. de Tonnancour, beaten from *Lythrumsalicaria* (1, PdTC); Saint-Lazare, 24.VI.2012, P. de Tonnancour, beaten from flowering *Brassica* sp. (1, PdTC).

**Distribution in Canada and Alaska.**ON, **QC**, **NB** (Bousquet et al. 2013).

***Bruchusbrachialis* Fåhraeus, 1839 †, new to Nova Scotia**

**Note.** The vetch bruchid, *B.brachialis*, is native to Europe and is a seed predator of *Vicia* spp. It was first collected in North America in NJ, DE, and MD in 1931 (Bottimer 1931) and by 1968 had spread to most of eastern and northwestern United States and southwestern Canada (Bottimer 1968). This is the first record for NS. Adults seek overwintering sites in protected places such as grain storage bins and under lichens and loose bark on trees. NS specimens emerged from trunk sections of red maple trees that had been girdled (the bark and top 2 cm of sapwood removed from circumference of the trunk near the ground) in spring of 2014, cut 23 February 2016, stored at -2 °C until 12 April 2016, and then incubated at 20 °C in emergence cages. The emergence date of 27 June 2016 may not accurately reflect the earliest date that adults became active because emergence cages were checked only twice after being set up: 27 June and 23 August 2016. In OR, Steinhauer (1959) observed the first adults of *B.brachialis* in early April and reported peak abundance of males in late May and females in late June. The girdling/felling/incubation of sections of red maple trees was intended as a way of detecting species of bark and wood boring beetles, but here we demonstrated that the method may also detect leaf feeders that overwinter under bark scales of trees. We collected several adults of the beech leaf-mining weevil, *Orchestesfagi* (L., 1758) from the same bolts.

**Specimen data. Nova Scotia**, **Halifax Co.**, Magazine Hill, 44.7143N, 63.6331W, emgd. 27.VI.2016 from red maple bolts coll. 23.II.2016, K. Van Rooyen & N. Higgins (9, AFC).

**Distribution in Canada and Alaska.**BC, ON, QC, **NS** (Bousquet et al. 2013).

#### Subfamily Donaciinae Kirby, 1837

***Donacia biimpressa* Melsheimer, 1847, new to New Brunswick**

**Specimen data. New Brunswick**, **Queens Co.**, Scotchtown, Grande Lake Meadows P.N.A., 45.8763N, 66.1822W, 16.VI.2013 (1), 17.VI.2013 (3), R.P. Webster // Lake shore / sand dune with red oak, sweeping vegetation (4, RWC).

**Distribution in Canada and Alaska.**MB, ON, QC, **NB** (Bousquet et al. 2013).

***Donacia limonia* C. Schaeffer, 1925, new to New Brunswick**

**Specimen data. New Brunswick, Queens Co.**, Upper Gagetown, bog adjacent to Hwy 2, 45.8316N, 66.2346W, 23.V.2006, R.P. Webster, coll. // Tamarack bog, treading *Carex* into water (1, RWC). **Restigouche Co.**, McDonald Road, 47.8333N, 68.2777W, 20.VI.2011, R. Webster & M. Turgeon // *Carex* marsh, sweeping vegetation (3, RWC); Dionne Brook P.N.A., 47.8981N, 68.3646W, 15.VI.2011 // Beaver flowage // pond, treading sedges into water (1, RWC). **Saint John Co.**, Musquash, 45.1856N, 66.3402W, 30.V.2008, R.P. Webster, coll. // *Carex* & cattail marsh, treading vegetation into water (1, RWC); Chance Harbour off Rt. 790, 45.1355N, 66.3672W, 12.V.2008, R.P. Webster, coll. // Shrubby cinquefoil fen, on *Carex* species (1, RWC). **Sunbury Co.**, Bull Pasture Bog, 46.0354N, 66.3358W, 21.VI.2013, R.P. Webster // Moss lawn bog with black spruce & tamarack on margin, sweeping vegetation on bog margin (2, RWC). **York Co.**, Charters Settlement, 45.8267N, 66.7343W, 14.V.2005, R.P. Webster, coll. // Margin of *Carex* marsh / fen in sphagnum & leaf litter at base of tree (1, RWC).

**Distribution in Canada and Alaska.**ON, QC, **NB** (Bousquet et al. 2013).

***Poeciloceraharrisii* (J.L. LeConte, 1851), new to Canada and New Brunswick**

**Note.***Poeciloceraharrisii* has been reported as far east as MA and NH in the Northeast (Riley et al. 2003) but has yet to be reported from ME (Majka et al, 2011). The NB record is a significant range extension to the northeast and represents the first occurrence of this species in Canada. Both specimens were captured in a calcareous fen with shrubby cinquefoil (*Dasiphorafruticosa* (L.) Rydb.). Little is known about the biology of the species other than an association with *Carex* (Riley et al. 2002).

**Specimen data. New Brunswick**, **York Co.**, Canterbury, Browns Mtn. Fen [now Eel River P.N.A.], 45.8967N, 67.6343W, 23.VI.2005, J. Edsall & R. Webster coll. // Calcareous fen, sweeping (1, RWC); same locality data but 23.VI.2014, R.P. Webster // Calcareous cedar fen with shrubby cinquefoil, sweeping vegetation with *Myricagale* & *Rhododendrongroenlandicum* (1, RWC).

**Distribution in Canada and Alaska. NB**

#### Subfamily Cassidinae Gyllenhal, 1813

***Chalepuswalshiiwalshii* (Crotch, 1873), new to New Brunswick**

**Note.***Chalepusw.walshii* was reported from ME by Majka et al. (2011). Larval hosts are grasses (Riley et al. 2003). The NB specimens were found on the adventive reed canary grass (*Phalarisarundinacea* L.).

In Bousquet et al. (2013), this species is incorrectly listed as *Chalepuswalshiwalshi* (Olivier, 1792). The correct name is *Chalepuswalshiiwalshii* (Crotch, 1873) (Yves Bousquet, Hume Douglas, and Patrice Bouchard, pers. comm., 2019).

**Specimen data. New Brunswick, York Co.**, Spednic Lake P.N.A., Palfrey Stream at East Brook Road, 45.6980N, 67.4982W, 13.VI.2018, R.P. Webster & M.-A. Giguère // Roadside, on grass (1, NBM); nr. Palfrey Stream at East Brook Road, 45.6987N, 67.4965W, 13.VI.2018, R.P. Webster & M.-A. Giguère // Roadside, on grass (2, RWC)

**Distribution in Canada and Alaska.**ON, **NB** (Bousquet et al. 2013).

***Chelymorphacassidea* (Fabricius, 1775)**

**Note.** Adults of *Chelymorphacassidea* were photographed in NB by Stuart Tingley at the Dunes de Bouchtouche in Bouchtouche on June 20 and June 30, 2015. These are the first records of this species in NB. Photographs of these specimens and others can be seen on the iNaturalist website at https://inaturalist.ca/observations?place_id=7587&taxon_id=216654

On August 2, 2019, Marie-Andrée Giguère and the first author visited the site and found both adults and pupae on bindweed (*Calystegiasepium* L.) growing in a salt marsh adjacent to sand dunes. Seven pupae were collected, and adults emerged from these pupae on August 5 and 6, 2019.

**Specimen data. New Brunswick, Kent Co.**, Dune de Bouctouche, 46.5212N, 64.6758W, 2.VIII.2019, R.P. Webster & M.-A. Giguère // Salt marsh adjacent to sand dune, on *Calystegiasepium* L. (3, NBM; 2, RWC); same data as above but ex. Pupa, emgd. 5–6.VIII.2019 (2, NBM; 5, RWC).

**Distribution in Canada and Alaska.**AB, SK, MB, ON, QC, **NB** (Bousquet et al. 2013).

#### Subfamily Galerucinae Latreille, 1802

***Chaetocnemahortensis* (Geoffroy, 1785) †, new to New Brunswick**

**Note.** Most specimens in the CNC from Canada determined as *Chaetocnemaborealis* R. White, 1996 are *C.hortensis* (Pentinsaari et al 2019). This species is native to the Palaearctic where it is widespread (Döberl 2010). It is adventive in Canada in BC and in eastern Canada in NF, LB, NS, and ON (Pentinsaari et al 2019). Here we report it for the first time from NB. Majka and LeSage (2010) reported *C.borealis* for the first time from NB, but it is possible that these specimens represent this adventive species. The specimen from Coxheath, Cape Breton Island, NS, illustrated as *C.borealis* in Majka and LeSage (2010) was dissected and is *C.hortensis* (Hume Douglas & Karine Savard, pers. comm.). This specimen exhibits the purple tone typical of most specimens from NB.

**Specimen data. New Brunswick**, **Kent Co.**, Kouchibouguac National Park, 46.8072N, 64.9082W, 21.V.2015, R.P. Webster // Alder swamp in mixed forest, in leaf litter (1, RWC). **Queens Co.**, W of Jemseg at “Trout Creek”, 45.8237N, 66.1225W, 6.IX.2007, R.P. Webster, coll. // Silver maple swamp, sweeping vegetation along margin of marsh (1, RWCD); Cranberry Lake P.N.A., 46.1125N, 65.6075W, 13–25.V.2011, R. Roy & V. Webster, coll. // Red oak forest, Lindgren funnel trap (1, RWC). **York Co.**, Charters Settlement, 45.8395N, 66.7391W, 17.VII.2014 (2), 27.VII.2006 (1), 27.VI.2007 (1), R.P. Webster, coll. // Mixed forest, m.v. light (4, RWC); same locality data and collector but 10.V.2007 // Mixed forest, in flight on warm (28 °C) sunny afternoon (1, RWC); Charters Settlement, 45.8263N, 66.7341W, 8.IX.2017, R.P. Webster // Pond margin / marsh, sweeping vegetation (1, RWC); Canterbury, trail to “Browns Mtn Fen”, 45.8964N, 67.6273W, 8.IX.2007, R.P. Webster, coll. // Mixed forest, sweeping roadside vegetation (1, RWC).

**Distribution in Canada and Alaska.**BC, ON, **NB**, NS, LB, NF (Pentinsaari et al. 2019).

***Chaetocnemairregularis* LeConte, 1857, new to New Brunswick**

**Specimen data. New Brunswick, York Co.**, Mazerolle Settlement, 45.8729N, 66.8311W, 28.IV.2006, R.P. Webster, coll. // Stream margin, in grass litter on muddy soil (1, RWC); Mazerolle Settlement, 45.8765N, 66.8260W, 8.VI.2008, R.P. Webster // Beaver meadow, sweeping vegetation along brook margin (1, RWC); Charters Settlement, 45.8348N, 66.7406W, 8.V.2018, R.P. Webster // Old brushy field, beating *Salix* catkins (1, NBM); Charters Settlement, 45.8263N, 66.7341W, 9.VI.2018, R.P. Webster // Pond margin / marsh, sweeping vegetation (7, RWC); Charters Settlement, 45.8451N, 66.7289W, R.P. Webster // Stream margin in marsh / alder swamp, sweeping cruciferous sp. (1, RWC); Spednic Lake P.N.A., near Pats Brook, 45.6209N, 67.4342W, 15.VIII.2017 (1), 15.VI.2018 (2), R.P. Webster // Freshwater marsh, sweeping vegetation (3, NBM); Spednic Lake P.N.A., Palfrey Stream at East Brook Rd., 45.6980N, 67.4982W, 13.VI.2018, R.P. Webster & M.-A. Giguère // Stream margin, sweeping vegetation (1, NBM); **Queens Co.**, W of Jemseg at “Trout Creek”, 45.8237N, 66.1225W, 6.IX.2007, R.P. Webster // Silver maple swamp, sweeping foliage along margin of marsh (1, RWC). **Sunbury Co.**, Burton, near Sunpoke Lake, 45.7662N, 66.5526W, 20.VI.2007, R.P. Webster, coll. // seasonally flooded marsh, sweeping vegetation (1, RWC).

**Distribution in Canada and Alaska.**NT, BC, AB, SK, MB, ON, QC, **NB** (Bousquet et al. 2013).

***Crepidoderadecora* Parry, 1986, new to New Brunswick**

**Note.***Crepidoderadecora* is externally very similar to *C.luminosa* Parry, 1986 and best distinguished by the shape of the male aedeagus and female spermathecae (Parry 1986). Both species were found on *Salix* catkins and foliage in NB.

**Specimen data. New Brunswick**, **Carleton Co.**, Red Brook Rd. at Meduxnekeag River, 46.1998N, 67.6989W, 7.VI.2018, R.P. Webster // River margin, sweeping vegetation (1 ♀ (dissected), RWC). **York Co.**, Charters Settlement, 45.8348N, 66.7406W, 6.V.2018 (3), 8.V.2018 (1), 17.V.2018 (3), R.P. Webster // Old brushy field, beating *Salix* catkins (2 ♀♀ (dissected), NBM; 3 ♂♂, 2 ♀♀ (dissected), RWC); near Thomaston Corner, 45.6171N, 67.0993W, 25.V.2018, R.P. Webster // Marsh, beating *Salix* foliage (3 ♀♀ (dissected), NBM; 2 ♂♂, 2 ♀♀ (dissected), RWC).

**Distribution in Canada and Alaska.**ON, QC, **NB** (Bousquet et al. 2013).

***Longitarsusmelanurus* (Melsheiner, 1847), new to New Brunswick**

**Specimen data. New Brunswick**, **York Co.**, Spednic Lake P.N.A., near Pats Brook, 45.6210N, 67.4342W, 15.VI.2018, R.P. Webster // Freshwater marsh with slow flowing stream with emergent vegetation, sweeping vegetation (3 (1 ♂ (dissected), RWC); Spednic Lake Prov. Park, 45.6183N, 67.4276W, 20.VI.2018, R.P. Webster // Marsh near Diggity Stream, treading *Carex* & grass (1, RWC).

**Distribution in Canada and Alaska.**MB, ON, QC, **NB** (Bousquet et al. 2013).

#### Subfamily Cryptocephalini Gyllenhal, 1813

***Diachuspallidicornis* (Suffrian, 1867), new to New Brunswick**

**New Record: New Brunswick**, **York Co.**, 16 km W of Tracy, off Rt. 645, 45.6854N, 66.8839W, 16.VIII–5.IX.2014, C. Alderson & V. Webster // Old red pine forest, Lindgren funnel trap (1, RWC).

**Distribution in Canada and Alaska.**ON, **NB** (Bousquet et al. 2013).

#### Family Anthribidae Billberg, 1820

**Subfamily Anthribinae Billberg, 1820**

***Anthribusnebulosus* Forster, 1770 †, new to Prince Edward Island and Quebec**

**Note.***Anthribusnebulosus* is a Palaearctic species that was intentionally introduced to VA in 1978–1979 as a biological agent against scale insects. It has since been recorded in southern New England (Hoebeke and Wheeler 1991) and adjacent regions of PA and NJ and, in Canada, from the region of Guelph, ON (Bouchard et al. 2017). Most specimens from QC were recovered from an intercept trap in a suburban residential area. Specimens from PE were captured in green or black Lindgren funnel traps set up either in the canopy of poplar (6) or 1 m high under trees (2).

**Specimen data. Prince Edward Island, Queens Co.**, Auburn, Auburn Demonstration Woodlot, 46.2882N, 63.9267W, 13.VI–3.VII.2018 (1), 2.VIII–13.IX.2018 (1), 4.VI–3.VII.2019 (3), 3.VII–14.VIII.2019 (2), 14.VIII–17.IX.2019 (1), C. Hughes // Mixed forest, green Lindgren funnel trap in canopy of poplar snag (1), green Lindgren funnel trap in canopy of poplar (5), low black Lindgren funnel trap (2) (2, AFC; 6, RWC). **Quebec, Agglomération de Montréal**, Sainte-Anne-de-Bellevue, 45.4091N, 73.9442W, 5.VII.2012, 15 h, P. de Tonnancour, beaten from *Ulmusamericana* + *Vitisriparia* (3, PdTC); Montréal, parc Zotique-Racicot, 45.543N, 73.690W, 6.VII.2015, ca. 14 h, beaten from *Fraxinus* sp., S. Dumont (1, SDC). **MRC de Deux-Montagnes**: Oka, Welcoming center, La Grande Baie, flight interception trap (white tulle fabric), 22.V.2018, R. Vigneault (1, RVC); **MRC de Vaudreuil-Soulanges**, Mont Rigaud, 45.4667N, 74.3258W, 12.VII.2014, 16 h, P. de Tonnancour, beaten from *Amelanchier* sp., rocky outcrop (1, PdTC); Notre-Dame-de-l’Île-Perrot, 45.3756N, 73.9448W, 26.VII.2015, 17 h, P. de Tonnancour, beaten from *Viburnumcassinoides* (1, PdTC); Terrasse-Vaudreuil, 45.3924N, 73.9922W, 7.V.2013, 17 h (1), 17.V.2013, 13–14 h (4), 30.V.2013, 14–15 h (1), 1.VI.2013,11 h (1), 12.V.2014, 14–18 h (2), 15.V.2014, 15 h (1), 20.V.2014, 18 h (1), 24.V.2014, 13 h (1), P. de Tonnancour, flight interception trap (white tulle fabric) (1, CCC; 3, CMNC; 2, CNC; 6, PdTC); same locality data and collector but 31.V.2013, 21–23 h 30, attracted to porch + UV lamps (2, CMNC; 2, PdTC); same locality data and collector but 25.VI.2019, 23 h 30, attracted to porch + UV + mercury vapor lamps (1, PdTC); Ville de l’Île-Perrot, 45.3969N, 73.9628W, 31.V.2015, 16 h, P. de Tonnancour, beaten from fruiting branches of *Fraxinuspennsylvanica* (1, PdTC); Terrasse-Vaudreuil, 45.3925N, 73.9923W, 11.X.2019, 15 h, resting between two freshly cut logs of *Fraxinuspennsylvanica* (1, PdTC).

**Distribution in Canada and Alaska.**ON, **QC**, **PE** (Bouchard et al. 2017).

#### Family Brentidae Billberg, 1820

**Subfamily Apioninae Schönherr, 1823**

**Rhopalapionlongirostre (Olivier, 1807) †**

**Note.** This distinctive looking adventive species was reported for the first time from NB on BugGuide.Net by Eric Knopf. See https://bugguide.net/node/view/1355652 for a photograph of this species. The first individual from Nasonworth, York, Co. was photographed on April 11, 2017. Another individual was photographed on July 10, 2017 https://bugguide.net/node/view/1401639. Determinations were verified by R.S. Anderson.

**Distribution in Canada and Alaska.**BC, SK, ON, QC, **NB**, NS (Bousquet et al. 2013).

***Fallapionmelanarium* (Gerstaecker, 1854), new to New Brunswick**

**Note.***Fallapionmelanarium* was reported from MA, NY, and ON south to TX by Downie & Arnett, Jr. (1996). It was not reported from ME by Majka et al. (2011). Host plants are *Bidens* spp. (beggar ticks) which occur in water or very moist situations (Tuttle 1954, Bright 1993). Most specimens from NB were swept from vegetation in marshes where *Bidens* was present.

**Specimen record. New Brunswick, Restigouche Co.**, Summit Lake, 47.7825N, 68.3199W, 7.VI.2011, R.P. Webster // Lake margin, *Carex* marsh, treading *Carex* hummocks and emergent vegetation (1, NBM; 2, RWC). **York Co.**, Charters Settlement, 45.8266N, 66.7365W, 2.VI.2007, R.P. Webster (1, NBM); Charters Settlement, 45.8263N, 66.7341W, 6.IX.2017 (4), 10.IX.2017 (8), R.P. Webster // Pond margin/marsh, sweeping vegetation (4, NBM; 8, RWC); 9.0 km W of Tracy, off Rt. 645, 45.6888N, 66.8004W, 22.V.2008, R.P. Webster, coll. // Sedge marsh, in *Carex* hummock (1, NBM).

**Distribution in Canada and Alaska.**ON, QC, **NB** (Bousquet et al. 2013).

***Stenopterapionmeliloti* (Kirby, 1808)** †

This adventive species associated with the Eurasian sweet clover (*Melilotusofficinalis* (L.) Lam.), as its specific name implies, was reported for the first time from NB on BugGuide.Net by Richard Migneault. See https://bugguide.net/node/view/503497 for a photograph of the specimen that was collected in Edmundston, Madawaska Co. on June 27, 2010. Another photo of an individual collected at the same locality on May 14, 2012 is shown at https://bugguide.net/node/view/736051. Determinations were verified by R.S. Anderson.

**Distribution in Canada and Alaska.**QC, **NB** (Bousquet et al. 2013).

#### Family Dryophthoridae Schönherr, 1825

**Subfamily Rhynchophorinae Schönherr, 1833**

***Sphenophorusaustralisaustralis* Chittenden, 1905, new to New Brunswick**

**Specimen data. New Brunswick**, **Albert Co.**, Waterside, Waterside Beach, 45.6282N, 64.8129W, 29.VI.2014, R.P. Webster // Salt marsh near tidal stream on sand in area with sparse *Spartina*. **York Co.**, Spednic Lake P.N.A., near Pats Brook, 45.6210N, 67.4342W, 15.VI.2018, R.P. Webster // Freshwater marsh, sweeping vegetation (1, RWC); Spednic Lake Prov. Park, 45.6183N, 67.4276W, 20.VI.2018, R.P. Webster // Marsh near Diggity Stream, treading *Carex* & grass (1, NBM).

**Distribution in Canada and Alaska.**ON, QC, **NB** (Bousquet et al. 2013).

#### Family Curculionidae Latreille, 1802

**Subfamily Curculioninae Latreille, 1802**

***Curculiorubidus* (Gyllenhal, 1835) †, new to Nova Scotia**

**Note.** This adventive weevil was first reported from Canada from QC and a BugGuide.Net record from ON (de Tonnancour et al. 2017). Most specimens from QC were beaten from foliage of gray birch (*Betulapopulifolia* Marshall) in late summer (de Tonnancour et al. 2017). This species will likely be found in NB.

**Specimen data. Nova Scotia, Halifax Co.**, Magazine Hill, 44.7143N, 63.6331W, 13–20.VIII.2018, K. Van Rooyen & J. Palmer // Hardwood forest, green Lindgren funnel trap in tree canopy (1, AFC).

**Distribution in Canada and Alaska.**ON, QC, **NS** (de Tonnancour et al. 2017).

***Curculiosulcatulus* (Casey, 1897), new to Nova Scotia**

**Specimen data. Nova Scotia, Halifax Co.**, Magazine Hill, 44.7143N, 63.6331W, 20–27.VIII.2018, K. Van Rooyen & J. Palmer // Hardwood dominated forest, green Lindgren funnel traps in tree canopy (4, AFC).

**Distribution in Canada and Alaska.**ON, QC, NB, **NS** (Bousquet et al. 2013).

***Ellescusbipunctatus* (Linnaeus, 1758), new to Nova Scotia**

**Note.** This species was first reported by Webster et al. (2016c) from NB, where it is common, and thus, its presence in NS is not unexpected.

**Specimen data. Nova Scotia, Halifax Co.**, Magazine Hill, 44.7143N, 63.6331W, 16.V.2016, K. Van Rooyen & N. Higgins (1, AFC).

**Distribution in Canada and Alaska.**MB, ON, QC, NB, **NS** (Bousquet et al. 2013, Webster et al. 2016c).

***Ellescusborealis* Carr, 1920, new to Quebec**

**Note.** The record of *E.borealis* from northwestern QC represents a significant range extension to the northeast. Like other *Ellescus* species, *E.borealis* is associated with *Salix* spp. It was found in abundance on smallfruit willow (*Salixbrachycarpa* Nuttall) growing on the eastern shore of James Bay.

**Specimen data. Quebec, Région administrative du Nord-du-Québec**, Longue-Pointe, 53.9752N, 79.0685W, 6.VIII.2018, 14 h, P. de Tonnancour, beaten from Salixbrachycarpavar.brachycarpa (11, PdTC); same locality and habitat data and collector but 7.VIII.2018, 12 h (59) (2, CCC; 5, CMNC; 3, CNC; 47, PdTC; 2, RVC).

**Distribution in Canada and Alaska.**AK, YT, AB, MB, **QC** (Bousquet et al. 2013).

***Mecinuspascuorum* (Gyllenhal, 1813)** †

This adventive weevil was reported for the first time from NB on BugGuide.Net by Richard Migneault. See https://bugguide.net/node/view/862006 for the illustration of the specimen which was collected in Edmundston, Madawaska Co. on June 23, 2013. This monophagous weevil feeds on the developing seeds of English plantain (*Plantagolanceolata* L.), also adventive in North America. Determination was verified by R.S. Anderson.

**Distribution in Canada and Alaska.**BC, ON, QC, **NB**, NS, PE (Bousquet et al. 2013).

***Orchestessteppensis* (Korotyaev, 2016) †, additional record from Nova Scotia.**

**Note.** As noted by Korotyaev (2016), this adventive species was misidentified as *Orchestesalni* (L., 1758) since its introduction in North America, and the distribution given in Bousquet et al. (2013) and the NS record of *O.alni* in Webster et al. (2016c) therefore applies to *O.steppensis*. An additional specimen of this species was reported from NS in 2019 and is reported below. This is the second record of this adventive species from the Maritime Provinces.

**Specimen data. Nova Scotia, Halifax Co.** Burnside Industrial Park, 42.87467N, 63.58778W, 9.VII.2019, K. Van Rooyen & C. Kostanowicz // Green Lindgren funnel trap in canopy of elm (*Ulmusamericana*) (1, CNC).

***Smicronyxamoenus* (Say, 1832), new to Quebec**

**Note.** The record from southern QC represents a significant eastern range extension in Canada, as this species was previously known only from SK and MB (Bousquet et al. 2013). Host records, although sparse, associate this species with Asteraceae (Anderson et al. 2014). Determination was made by R.S. Anderson.

**Specimen data. Quebec, MRC de Vaudreuil-Soulanges**, Terrasse-Vaudreuil, 45.3924N, 73.9923W, 14.VIII.2018, 1–2 h, P. de Tonnancour, attracted to porch + mercury vapor lamps (1, PdTC).

**Distribution in Canada and Alaska.**SK, MB, **QC** (Bousquet et al. 2013).

#### Subfamily Bagoinae C.G. Thomson, 1859

***Bagousmagister* LeConte, 1876, new to Nova Scotia**

**Specimen data. Nova Scotia, Halifax Co.**, Magazine Hill, 44.7143N, 63.6331W, 4.VII.2016, K. Van Rooyen & N. Higgins // hardwood dominated forest, purple Lindgren funnel in understory (1, AFC).

**Distribution in Canada and Alaska.**ON, QC, **NS** (Bousquet et al. 2013, de Tonnancour et al. 2017).

#### Subfamily Baridinae Schönherr, 1836

***Plesiobarisalbilata* (LeConte, 1876), new to Canada, New Brunswick, and Quebec**

Specimens recorded as *Plesiobarisdisjuncta* Casey, 1892 in Webster et al. (2012a) were reviewed following examination of the holotype of this species by R.S. Anderson in February 2020. These records from NB and QC were found to refer to *Plesiobarisalbilata* (LeConte, 1876), a species not previously recorded in Canada, thus *P.disjuncta* should be removed from the Canadian, NB and QC faunal lists.

**Distribution in Canada and Alaska. QC, NB**.

#### Subfamily Ceutorhynchinae Gistel, 1848

***Ceutorhynchusoregonensis* Dietz, 1896**

This weevil was reported for the first time from NB on BugGuide.Net by Richard Migneault. See https://bugguide.net/node/view/736516 for the illustration of the specimen that was collected in Edmundston, Madawaska Co. on June 12, 2012. The determination was confirmed by R.S. Anderson.

**Distribution in Canada and Alaska.**YT, BC, AB, MB, ON, QC, **NB**, NS (Bousquet et al. 2013).

***Prorutidosomadecipiens* (LeConte, 1876)**

This weevil was reported for the first time from NB on BugGuide.Net by Richard Migneault. See https://bugguide.net/node/view/1029494 for the illustration of the specimen that was collected in Edmundston, Madawaska Co. on May 29, 2014. The determination was confirmed by R.S. Anderson.

**Distribution in Canada and Alaska.**AK, YT, BC, AB, SK, MB, ON, QC, **NB**, PE (Bousquet et al. 2013).

#### Subfamily Cossoninae Schönherr, 1825

***Phloeophaguscanadensis* Van Dyke, 1927, new to Nova Scotia**

**Specimen data. Nova Scotia, Halifax Co.**, Magazine Hill, 44.7143N, 63.6331W, 27.VI.2016, coll., K. Van Rooyen & H. Higgins (1, AFC); same locality data but 4–11.VII.2016, K. Van Rooyen & N. Higgins // Hardwood dominated forest, purple Lindgren funnel trap in understory (1, AFC).

**Distribution in Canada and Alaska.**AK, BC, AB, SK, MB, QC, NB, **NS** (Bousquet et al. 2013).

#### Subfamily Entiminae Schönherr, 1823

***Polydrususimpressifrons* Gyllenhal, 1834 †, new to Prince Edward Island**

**Specimen data. Prince Edward Island, Kings Co.**, Valleyfield, Valleyfield Demonstration Woodlot, 46.1356N, 62.7198W, 12.VI–3.VII.2018, C. Hughes // Green Lindgren funnel trap in canopy of poplar (1, RWC).

**Distribution in Canada and Alaska.**MB, ON, QC, NB, NS, **PE** (Bousquet et al. 2013).

#### Subfamily Lixinae Schönherr, 1823

***Larinuscarlinae* (Olivier, 1807)** †

This adventive weevil was reported for the first time from NB on BugGuide.Net by Richard Migneault. See https://bugguide.net/node/view/736349 for the illustration of the specimen that was collected in Edmundston, Madawaska Co. on June 12, 2012. This species was listed as *Larinusplanus* (Fabricius, 1792) by Bousquet et al. (2013) and is still reported as such on BugGuide.Net. Gültekin and Alonso-Zarazaga (2015) showed that the correct name is *Larinuscarlinae* (Olivier, 1807). Determination was verified by R.S. Anderson.

**Distribution in Canada and Alaska.**BC, AB, ON, QC, **NB**, NS (Bousquet et al. 2013).

#### Subfamily Mesoptiliinae Lacordaire, 1863

***Magdalisalutacea* LeConte, 1878, new to Nova Scotia**

**Specimen data. Nova Scotia, Halifax Co.**, Magazine Hill, 44.7143N, 63.6331W, 2.VII.2015, K. Van Rooyen & T. Nelson (3, AFC).

**Distribution in Canada and Alaska.**AK, YT, NT, BC, AB, SK, QC, NB, **NS** (Bousquet et al. 2013).

***Magdalishispoides* LeConte, 1876, new to Nova Scotia**

**Specimen data. Nova Scotia, Halifax Co.**, Magazine Hill, 44.7143N, 63.6331W, 4.VII.2016 (2), 11.VII.2016 (1), Coll., K. Van Rooyen & N. Higgins (3, AFC).

**Distribution in Canada and Alaska.**YT, BC, AB, ON, QC, NB, **NS**, NF (Bousquet et al. 2013).

#### Subfamily Scolytinae Latreille, 1804

***Pityophthorusramiperda* Swaine, 1917, new to New Brunswick**

**Specimen data. New Brunswick, Kent Co.**, Kouchibouguac N.P., 46.8072N, 64.9100W, 27.V–12.VI.2015, C. Alderson & V. Webster // Jack pine forest, Lindgren funnel trap 1 m high (2, RWC). **York Co.** Odell Park, 45.9508N, 66.6723W, 19.V–3.VI.2015, C. Alderson & V. Webster // Old mixed forest, Lindgren funnel trap in canopy of hardwood (1, RWC).

**Distribution in Canada and Alaska.**ON, QC, **NB**, NS (Bousquet et al. 2013).

***Pseudopityophthorusasperulus* (LeConte, 1868), new to Canada and Nova Scotia**

**Note.** This species differs from the much more common *Pseudopityophthorusminutissimus* (C.C.A. Zimmerman, 1868) by being more slender, and having elytral striae with minute punctures in fairly definite rows. In *P.minutissimus* there is little evidence of strial rows (Bright 1976). Bright (1976) predicted this species might be found in NB as it is known from ME. Its presence in NS is not unexpected.

**Specimen data. Nova Scotia, Halifax Co.**, Magazine Hill, 44.7143N, 63.6331W, 16.V.2016, K. Van Rooyen & N. Higgins // Hardwood dominated forest, purple Lindgren funnel trap in tree canopy (1, AFC); same locality, habitat and collectors but 17–30.V.2016, green Lindgren funnel trap 1 m above ground (1, AFC); same locality but 3–10.VI.2018, K. Van Rooyen & J. Palmer // green Lindgren funnel traps 1m above ground (2), in tree canopy (1). (3, AFC).

**Distribution in Canada and Alaska. NS**.

***Hylastesopacus* Erichson, 1836 †, new to Nova Scotia and Prince Edward Island**

**Note.** Since its initial discovery in North America, in the state of NY in 1989 (Wood, 1992), this adventive Palaearctic species has rapidly extended its distribution and now occurs in several Canadian provinces and states in the United States. Its main hosts are *Pinus* spp.

**Specimen data. Nova Scotia, Halifax Co.**, Magazine Hill, 44.7143N, 63.6331W, 16.V.2016, coll., K. Van Rooyen & N. Higgins (1, AFC). **Prince Edward Island, Kings Co.**, Valleyfield, Valleyfield Demonstration Woodlot, 46.1385N, 62.7194W, 7.V–4.VI.2019, C. Hughes // Green Lindgren funnel trap in canopy of poplar (1, AFC). **Queens Co.**, Brookvale, Brookvale Demonstration Woodlot, 46.2920N, 63.4052W, 13.VI–3.VII.2018, 4.VI–3.VII.2019, C. Hughes // Lindgren funnel traps 1 m high (2, AFC).

**Distribution in Canada and Alaska.**BC, ON, QC, NB, **NS, PE** (Bousquet et al. 2013).

***Hylurgopspalliatus* (Gyllenhal, 1813) †, new to Canada and New Brunswick**

**Note.** This Eurasian species was one of the most commonly intercepted species in inspections of wood packaging material at United States ports between 1985 and 2005 (Haack 2006). Its first North American records were from PA (Haack 2001) and later NY and OH (Hoebeke and Acciavatti 2006). There have been no reports of damage or hosts in the United States (Haack 2006). The specimen reported below was determined by Donald Bright in 2020 and represents a new record for Canada.

**Specimen data. New Brunswick, York Co.**, Fredericton, U.N.B. Woodlot, 45.9206N, 66.6520W, 31.V–14.VI.2013, C. Alderson & V. Webster // Mature mixed forest, Lindgren funnel trap 2 m high (1, RWC).

**Distribution in Canada and Alaska. NB**.

***Hylastinusobscurus* (Marsham, 1802) †, new to New Brunswick**

**Note.** This adventive species is known as the clover root borer and is sometimes a serious pest of clover and alfalfa in the eastern United States (Bright 1976). A specimen (determination confirmed by RPW) of this species from Edmundston, Madawaska Co. was photographed by Richard Migneault on June 18, 2019. See https://bugguide.net/node/view/1785474.

**Specimen data. New Brunswick, York Co.**, Crabbe Mountain, 46.12115N, 67.10524W, 6–27.VI.2018, C. Alderson & V. Webster // Hardwood forest, black Lindgren funnel trap in open tree canopy (1), green Lindgren funnel trap on edge of tree canopy (1) (2, AFC); Keswick Ridge, 45.99618N, 66.87813W, 11–29.VI.2018, C. Alderson & V. Webster // Hardwood forest, black Lindgren funnel trap in understory (1, AFC).

**Distribution in Canada and Alaska.**BC, ON, QC, **NB**, NS (Bousquet et al. 2013).

***Heteroboripsseriatus* (Blandford, 1894) †, new to Canada and Nova Scotia**

**Note.** This specimen was determined by R.J. Rabaglia in 2019 and represents a new record for Canada. This Asian species was first detected in North America in MA in 2005 (Hoebeke and Rabaglia 2008) and subsequently in ME in 2009 and PA in 2011, using semiochemical-baited black funnel traps as part of the US Forest Services Early Detection Rapid Response (EDRR) trapping surveillance program (Rabaglia et al. 2019). Mandelshtam et al. (2019) recently removed the genus *Heteroborips* from synonymy with *Xyleborus* and placed *Xyleborusseriatus* Blandford in the genus *Heteroborips*.

**Specimen data. Nova Scotia, Halifax Co.**, Magazine Hill, 44.7143N, 63.6331W, 16–23.VII.2018, K. Van Rooyan & J. Palmer // Hardwood dominated forest, black Lindgren funnel trap in understory (1, AFC).

**Distribution in Canada and Alaska. NS**

***Xyleborinussaxesenii* (Ratzeburg, 1837) †, new to Prince Edward Island**

**Note.** Since its initial discovery in the USA (NY) in 1890 and in Canada (BC) in 1928, this highly polyphagous species has extended its range to more than 30 states and to ON, QC, and the Maritimes in Canada (Klimaszewski et al. 2010).

**Specimen data. Prince Edward Island, Kings Co.**, Valleyfield, Valleyfield Demonstration Woodlot, 46.1385N, 62.7194W, 4.VI–3.VII.2019, 3.VII–13.VIII.2019, C. Hughes // Lindgren funnel traps 1 m high (2, AFC); New Harmony, New Harmony Demonstration Woodlot, 46.3914N, 62.2021W, 3.VII–13.VIII.2019, C. Hughes // Lindgren funnel trap 1 m high (1, AFC).

**Distribution in Canada and Alaska.**BC, ON, QC, NB, NS, **PE** (Bousquet et al. 2013).

***Xylosandrusgermanus* (Blandford, 1894) †, new to Prince Edward Island and New Brunswick**

**Note.** This ambrosia beetle is native to Japan, Korea, the Kuril Islands, Vietnam, China, and Taiwan, and is adventive in Central Europe and North America. In Europe, it was first detected in Germany in 1951 and has spread to Austria, Belgium, France, Italy, and Switzerland (Ranger et al. 2010). It was first detected in North America in NY in 1932 and has since become established over much of the United States (Rabaglia et al. 2006). It was first reported in Canada from ON in 1987 (Bright 1989). It is highly polyphagous, infesting several families of coniferous and broadleaf trees but prefers the latter. Common hosts include black walnut (*Juglansnigra* L.), American beech, and maples (*Acer* spp.) (Weber and McPherson 1983; Ranger et al. 2010). It typically colonizes stressed trees but can be a significant pest of tree nurseries (Olivier and Mannion 2001) and apple orchards (Agnello et al. 2017) in the United States. Adults are attracted to ethanol (Miller and Rabaglia 2009; Ranger et al. 2010).

**Specimen data. New Brunswick, Queens Co.**, Gagetown, 45.75162N, 66.18655W, 27VII–14.VIII.2017, C. Alderson & V. Webster // Old mixed forest with *Quercusrubra*, Lindgren funnel trap in tree canopy (1, AFC). **York Co.** Kingsclear, 45.9458N, 66.7948W, 8–21.VII.2017, C. Alderson & V. Webster // Mixed forest, Purple Lindgren funnel trap 1 m above ground. (1, AFC). **Prince Edward Island, Queens Co.**, Brookvale, Brookvale Demonstration Woodlot, 46.2920N, 63.4052W, 4.VI–3.VII.2019, C. Hughes // White panel trap (1, AFC).

**Distribution in Canada and Alaska.**BC, ON, QC, **NB**, NS, **PE** (Bousquet et al. 2013).

## Footnotes

Newfoundland and Labrador are each treated separately under the heading Distribution in Canada and Alaska.

## References

[B1] Agnello AM Breth DI Tee EM Cox KD Villani SM Ayer KM Wallis AE Donahue DJ Combs DB Davis AE Neal JA English-Loeb FM (2017) *Xylosandrusgermanus* (Coleoptera: Curculionidae: Scolytinae) occurrence, fungal associations, and management trials in New York apple orchards. Journal of Economic Entomology 110: 2149–2164. 10.1093/jee/tox189 29048587

[B2] Anderson RS Bouchard P Douglas H (2014) Weevils (Coleoptera: Dryophthoridae, Brachyceridae, Curculionidae) of the Prairies Ecozone in Canada. In: Giberson DJ Cárcamo HA (Eds) Arthropods of Canadian Grasslands (Volume 4): Biodiversity and Systematics Part 2.Biological Survey of Canada, 143–167.

[B3] Ashbee HV Marshall SA Alarie Y (2017) Haliplidae of eastern Canada. Canadian Journal of Arthropod Identification 32: 1–80.

[B4] Barney RJ LeSage L Savard K (2013) *Pachybrachis* (Coleoptera, Chrysomelidae, Cryptocephalinae) of eastern Canada. ZooKeys 332: 95–175. 10.3897/zookeys.332.4753 PMC3804768 24163583

[B5] Bottimer LJ (1931) A vetch bruchid established in the Middle Atlantic States. Insect Pest Survey Bulletin 11: 347, 955–435.

[B6] Bottimer LJ (1968) Notes on Bruchidae of America north of Mexico with a list of world genera. The Canadian Entomologist 100: 1009–1049. 10.4039/Ent1001009-10

[B7] Bouchard P Webster RP Klimaszewski J (2016) The Coleoptera of New Brunswick and Canada: Providing baseline biodiversity and natural history data [Special issue]. ZooKeys 573: 1–512.

[B8] Bouchard P Smith ABT Douglas H Gimmel ML Brunke AJ Kanda K (2017) Chapter 11, Biodiversity of Coleoptera. In: Foottit RG Adler PH (Eds) Insect Biodiversity: Science and Society.Vol. 1, Second Edition. John Wiley & Sons, Ltd., West Sussex, UK, 337–417. 10.1002/9781118945568.ch11

[B9] Bousquet Y (1990) A review of the North American species of *Rhizophagus* Herbst and a revision of the Nearctic members of the subgenusAnomophagus Reitter (Coleoptera: Rhizophagidae). The Canadian Entomologist 122: 131–171. 10.4039/Ent122131-1

[B10] Bousquet Y (1991) Checklist of beetles of Canada and Alaska. Research Branch, Agriculture Canada, Ottawa. Cat. no. A43-1861/1991E, vi + 430 pp.

[B11] Bousquet Y (2010) Illustrated identification guide to adults and larvae of northeastern North American ground beetles (Coleoptera: Carabidae). Pensoft, Sofia-Moscow, 562 pp.

[B12] Bousquet Y Bouchard P (2014) Review of the species of *Paratenetus* Spinola inhabiting America, north of Mexico (Coleoptera, Tenebrionidae). ZooKeys 415: 23–51. 10.3897/zookeys.415.6524 PMC4089819 25009423

[B13] Bousquet Y Bouchard P Davies AE Sikes D (2013) Checklist of beetles (Coleoptera) of Canada and Alaska. Pensoft Series Faunistica No. 109, Sofia-Moscow, 402 pp. 10.3897/zookeys.360.4742 PMC3867111 24363590

[B14] Bousquet Y Laplante S (2000) Présence de *Caerosternusamericanus* (J.E. LeConte) et de *Xestipygegeminatum* (J.E. LeConte) (Coleoptera: Histeridae) au Québec. Fabreries 24 [1999]: 73–77.

[B15] Bousquet Y Laplante S (2006) The Insects and Arachnids of Canada, Part 24. ColeopteraHisteridae. NRC Research Press, Ottawa, Ontario, Canada, 485 pp.

[B16] Bousquet Y Laplante S Hammond HEJ Wagner DW (2017) Cerambycidae (Coleoptera) of Canada and Alaska: Identification guide with nomenclatural, taxonomic, distributional, host-plant, and ecological data. Nakladatelství Jan Farkač, Prague, 300 pp.

[B17] Bousquet Y Thomas DB Bouchard P Smith AD Aalbu RL Johnston MA Steiner WE Jr (2018) Catalogue of Tenebrionidae (Coleoptera) of North America. ZooKeys 728: 1–455. 10.3897/zookeys.728.20602 PMC5799738 29416389

[B18] Bright DE Jr (1976) The Bark Beetles of Canada and Alaska. Coleoptera: Scolytidae. The Insects and Arachnids of Canada. Part 2. Agriculture Canada, Ottawa, 241 pp.

[B19] Bright DE (1989) *Xylosandrusgermanus* (Blandford), an ambrosia beetle new to Canada. The Canadian Agricultural Insect Pest Review 66 [1988]: 93–94.

[B20] Bright DE (1993) The weevils of Canada and Alaska: Volume 1. Coleoptera: Curculionoidea, excluding Scolytidae and Curculionidae. The insects and arachnids of Canada. Part 21. Agriculture Canada, Ottawa, 217 pp.

[B21] Bright DE Skidmore RE (2002) A Catalog of Scolytidae and Platypodidae (Coleoptera): Supplement 2 (1995–1999). National Research Council Research Press, Ottawa, 523 pp.

[B22] Brousseau P-M Gravel D Handa IT (2014) The first record in Canada of *Onthophiluspluricostatus* LeConte (Coleoptera: Histeridae) and a new mention for the rare species *Lordithonniger* (Gravenhorst) (Coleoptera: Staphylinidae). The Coleopterists Bulletin 68(2): 343–344. 10.1649/0010-065X-68.2.343

[B23] Brown WJ (1950) The extralimital distribution of some species of Coleoptera. The Canadian Entomologist 82: 197–205. 10.4039/Ent82197-10

[B24] Dearborn RG Nelson RE Donahue C Bell RT Webster RP (2014) The ground beetle (Coleoptera: Carabidae) fauna of Maine, USA. The Coleopterists Bulletin 68: 441–599. 10.1649/072.068.0317

[B25] de Tonnancour P Anderson RS Bouchard P Chantal C Dumont S Vigneault R (2017) New Curculionoidea (Coleoptera) records for Quebec, Canada. ZooKeys 681: 95–117. 10.3897/zookeys.681.12469 PMC5523881 28769721

[B26] Döberl M (2010) Alticinae. In: Löbl I Smetana A (Eds) Catalogue of Palaearctic Coleoptera (Vol 6).Apollo Books, Stenstrup, 491–563.

[B27] Douglas H Bouchard P Anderson RS de Tonnancour P Vigneault R Webster RP (2013) New Curculionoidea (Coleoptera) records for Canada. ZooKeys 309: 13–48. 10.3897/zookeys.309.4667 PMC3689125 23794927

[B28] Downie NM Arnett RH Jr (1996) The beetles of northeastern North America, Vol. 1 & 2, Sandhill Crane Press, Gainesville Florida, 1721 pp.

[B29] Dumont S de Tonnancour P (2019) First records of *Larinusturbinatus* Gyllenhal (Coleoptera: Curculionidae: Lixinae) in Canada. The Coleopterists Bulletin 73: 828–830. 10.1649/0010-065X-73.4.828

[B30] Enushchenko IV (2017) Five new species of the tribe Gyrophaenina Kraatz 1830 (Coleoptera: Staphylinidae: Aleocharinae) from the southern part of the United States. Zootaxa 4504: 209–224. 10.11646/zootaxa.4504.2.3 30486025

[B31] Esser J (2016) Über die Identität von *Cryptophilusinteger* (Heer, 1841) (Coleoptera: Erotylidae). Entomologische Nachrichten und Berichte 60: 213–218.

[B32] Esser J (2017) On the Nearctic *Cryptophilus* Reitter, 1874 (Coleoptera: Erotylidae). Linzer Biologische Beiträge 49: 1133–1137.

[B33] Esser J (2018) Notes on taxonomy and distribution of *Cryptophagusjakowlewi* Reitter, 1888 (Coleoptera: Cryptophagidae) with remarks on further species. Linzer Biologische Beiträge 50: 245–253.

[B34] Fauvel A (1889) Liste des coléoptères communs à l’Europe et à l’Amérique du Nord. D’après le catalogue de M. J. Hamilton. Avec remarques et additions. Revue d’Entomologie 8: 92–174.

[B35] Frank JH (1975) A revision of the New World species of the genus *Erichsonius* Fauvel (Coleoptera: Staphylinidae). The Coleopterists Bulletin 29: 177–203.

[B36] Frank JH (1981) A new *Erichsonius* species from Arizona with discussion on phylogeny within the genus (Coleoptera: Staphylinidae). The Coleopterists Bulletin 35: 97–106.

[B37] Gordon RD (1976) The Scymnini (Coleoptera: Coccinellidae) of the United States and Canada: key to genera and revision of *Scymnus*, *Nephus*, and *Diomus*. Bulletin of the Buffalo Society of Natural Science. No. 28: 362 pp.

[B38] Gordon RD (1985) The Coccinellidae (Coleoptera) of America north of Mexico. Journal of the New York Entomological Society 93: 1–912.

[B39] Gültekin L Alonso-Zarazaga MA (2015) A review of the Palaearctic species of *Larinus* Dejean (Coleoptera: Curculionidae) in C. J. Schoenherr collection: Nomenclature and lectotype designations. Journal of Insect Biodiversity 3(9): 1–26. 10.12976/jib/2015.3.9

[B40] Haack RA (2001) Intercepted Scolytidae (Coleoptera) at U.S. ports of entry: 1985–2000. Integrated Pest Management Review 6: 253–282. 10.1023/A:1025715200538

[B41] Haack RA (2006) Exotic bark- and wood-boring Coleoptera in the United States: recent establishments and interceptions. Canadian Journal of Forest Research 36: 269–288. 10.1139/x05-249

[B42] Hardy M (2014) Guide d’identification des scarabées du Québec (Coleoptera: Scarabaeoidea). Entomofaune du Québec Inc., Saguenay, 166 pp.

[B43] Háva J Nei I (2016) New records of Megatoma (Pseudohadrotoma) graeseri (Reitter, 1887) (Coleoptera: Dermestidae: Megatominae) from Canada. Onychium 12: 135–136. 10.11646/zootaxa.3731.4.11 25277594

[B44] Herms DA McCullough DG (2014) Emerald ash borer invasion of North America: History, biology, ecology, impacts, and management. Annual Review of Entomology 59: 12–30. 10.1146/annurev-ento-011613-162051 24112110

[B45] Hoebeke ER Acciavatti RE (2006) *Hylurgopspalliatus* (Gyllenhal) (Coleoptera: Curculionidae: Scolytinae), an Eurasian bark beetle new to North America (Coleoptera: Curculionidae: Scolytinae). Proceedings of the Entomological Society of Washington 108: 267–273.

[B46] Hoebeke ER Rabaglia RJ (2008) *Xyleborusseriatus* Blandford (Coleoptera: Curculionidae: Scolytinae), an Asian ambrosia beetle new to North America. Proceedings of the Entomological Society of Washington 110: 470–476. 10.4289/07-048.1

[B47] Hoebeke ER Wheeler AG Jr (1991) *Anthribusnebulosus*, a Eurasian scale predator in the eastern United States (Coleoptera: Anthribidae): Notes on biology, recognition, and establishment. Proceedings of the Entomological Society of Washington 93: 45–50.

[B48] Hoebeke ER Wheeler AG Jr (1996) Adventive lady beetles (Coleoptera: Coccinellidae) in the Canadian Maritime Provinces, with new eastern U.S. records of *Harmoniaquadripunctata*. Entomological News 107: 281–290.

[B49] Hughes CC Johns RC Sweeney JD (2014) A technical guide to installing beetle traps in the upper crown of trees. Journal of the Acadian Entomological Society 10: 12–18.

[B50] Jendek E Grebennikov VG Bocak L (2015) Undetected for a century: Palaearctic *Agrilusribesi* Schaefer (Coleoptera: Buprestidae) on currant in North America, with adult morphology, larval biology and DNA barcode. Zootaxa 4034: 112–126. 10.11646/zootaxa.4034.1.5 26624433

[B51] Kingsolver JM (2004) Handbook of the Bruchidae of the United States and Canada (Insecta, Coleoptera). US Department of Agriculture. Technical Bulletin No. 1912, 2 vol., 636 pp.

[B52] Klimaszewski J Hoebeke R Godin B Davies A Perry KI Bourdon C Winchester N (in press) Aleocharine rove beeetles of British Columbia: a hotspot of Canadian biodiversity (Coleoptera, Staphylinidae). Springer Nature, Cham, Switzerland, xvi + 621 pp.

[B53] Klimaszewski J Langor DW Hammond HEJ Pelletier G Bousquet Y Bourdon C Webster RP Borowiec L Scudder GGE Majka CG (2015) Synopsis of adventive species of Coleoptera (Insecta) recorded from Canada. Part 3: Cucujoidea. Pensoft, Sofia-Moscow, Pensoft Series Faunistica No. 113, 171 pp.

[B54] Klimaszewski J Langor D Majka CG Bouchard P Bousquet Y LeSage L Smetana A Sylvestre P Pelletier G Davies A DesRochers P Goulet H Webster R Sweeney J (2010) Review of adventive species of Coleoptera (Insecta) recorded from eastern Canada. Pensoft, Sofia-Moscow, Pensoft Series Faunistica No. 94, 272 pp.

[B55] Klimaszewski J Webster RP Davies A (2017) Genus *Hydrosmecta* C.G. Thomson: a review of species occurring in eastern Canada (Coleoptera, Staphylinidae, Aleocharinae). Insecta Mundi 0593: 1–17.

[B56] Klimaszewski J Webster RP Davies A Bourdon C (2018a) Description of Hydrosmectomorpha Klimaszewski and Webster, a new subgenus of Atheta C.G. Thomson, with three new Canadian species (Coleoptera: Staphylinidae: Aleocharinae) Insecta Mundi 0648: 1–12.

[B57] Klimaszewski J Webster RP Langor DW Brunke A Davies A Newton AF Bourdon C Labrecque M Dorval JA Frank JH (2018b) Aleocharinae rove beetles of eastern Canada (Coleoptera, Staphylinidae, Aleocharinae): a glimpse of megadiversity. Springer Nature Switzerland AG., xvi + 902 pp. 10.1007/978-3-319-77344-5

[B58] Knopf E Gilmore W (2018) The first recorded occurrences of *Platydracusimmaculatus* (Mannerheim) and *Ocypusnitens* (Schrank), (Coleoptera; Staphylinidae), in New Brunswick, Canada. Journal of the Acadian Entomological Society 14: 25–27.

[B59] Korotyaev BA (2016) New data on the changes in the abundance and distribution of several species of beetles (Coleoptera) in European Russia and the Caucasus. Entomological Review 96: 620–630. 10.1134/S0013873816050080

[B60] Kovarik PW Caterino MS (2001) Family 15, Histeridae Gyllenhal, 1808. [p. 212–227]. In: Arnett RH, Thomas MC Jr. (Eds) American Beetles, Volume 1. Archostemata, Myxophaga, Adephaga, Polyphaga: Staphyliniformia. CRC Press, Boca Raton, Florida, xv + 443 p.

[B61] Larson DJ Alarie Y Roughley RE (2000) Predaceous diving beetles (Coleoptera: Dytiscidae) of the Nearctic region, with emphasis on the fauna of Canada and Alaska. NRC Research Press, Ottawa, xiv + 982 pp.

[B62] Lebel M Dumont S Racine M (2019) Nouvelles mentions de trois espèces de Cleridae (Coleoptera) pour le Québec. Le Naturaliste Canadian 143(2): 12–17. 10.7202/1060051ar

[B63] Liljeblad E (1945) Monograph of the family Mordellidae (Coleoptera) of North America, north of Mexico. Miscellaneous Publications, University of Michigan, Museum of Zoology, No. 62, 229 pp.

[B64] Lindgren BS (1983) A multiple funnel trap for scolytid beetles (Coleoptera). The Canadian Entomologist 115: 299–302. 10.4039/Ent115299-3

[B65] Lisberg AE (2003) Taxonomic changes for fifteen species of North American Mordellidae (Coleoptera). Insecta Mundi 17: 191–194.

[B66] Majka CG Chandler DS Donahue CP (2011) Checklist of the beetles of Maine, USA. Empty Mirrors Press, Halifax, Nova Scotia, 328 pp.

[B67] Majka CG Gimmel ML Langor D (2008) The Phalacrididae (Coleoptera: Cucujoidea) of Canada: new records, distribution, and bionomics with a particular focus on the Atlantic Canadian fauna. ZooKeys 2: 209–220. 10.3897/zookeys.2.16

[B68] Majka CG Klimaszewski J (2004) *Phloeocharissubtilissima* Mannerheim (Staphylinidae: Phloeocharinae) and *Cephenniumgallicum* Ganglbauer (Scydmaenidae) new to North America: a case study in the introduction of exotic Coleoptera to the port of Halifax, with new records of other species. Zootaxa 781: 1–15. 10.11646/zootaxa.781.1.1

[B69] Majka CG LeSage L (2010) *Chaetocnema* flea beetles (Coleoptera: Chrysomelidae, Alticini) of the Maritime Provinces of Canada. Journal of the Acadian Entomological Society 6: 34–38.

[B70] Majka CG McCorquodale DB (2006) The Coccinellidae (Coleoptera) of the Maritime Provinces of Canada: new records, biogeographic notes, and conservation concerns. Zootaxa 1154: 49–68. 10.11646/zootaxa.1154.1.5

[B71] Majka CG McCorquodale DB (2010) Lady beetles (Coleoptera: Coccinellidae) of the Atlantic Maritime Ecozone. In: McAlpine DF Smith LM (Eds) Assessment of Species diversity in the Atlantic Maritime Ecozone.National Research Council Press, Ottawa, Ontario, 439–452.

[B72] Mandelshtam MY Petrov AV Smith AM Cognato AI (2019) Resurrection of *Heteroborips* Reitter, 1913 (Coleoptera: Curculionidae: Scolytinae) from synonymy with *Xyleborus* Eichhoff, 1864. The Coleopterists Bulletin 73: 387–394. 10.1649/0010-065X-73.2.387

[B73] Matta JF Wolfe GW (1981) A revision of the subgenusHeterosternuta Strand of *Hydroporus* Clairville (Coleoptera: Dytiscidae). The Pan-Pacific Entomologist 57: 176–219.

[B74] McAlpine DF Migneault R Webster RP (2018) *Coleomegillamaculatalengi* Timberlake, 1943 (Coleoptera: Coccinellidae), a native North American lady beetle new to Maritime Canada. Journal of the Acadian Entomological Society 14: 8–10.

[B75] McNamara J (1992) The first Canadian records of Scymnus (Pullus) suturalis Thunberg (Coleoptera: Coccinellidae). The Coleopterists Bulletin 46: 359–360.

[B76] Miller DR Rabaglia RJ (2009) Ethanol and (-)-α-pinene: Attractant kairomones for bark and ambrosia beetles in the Southeastern US. Journal of Chemical Ecology 35: 435–448. 10.1007/s10886-009-9613-9 19294470

[B77] Oliver J Mannion CM (2001) Ambrosia beetle (ColeopteraScolytidae) species attacking chestnut and captured in ethanol-baited traps in middle Tennessee. Journal of Economic Entomology 30: 909–918. 10.1603/0046-225X-30.5.909

[B78] Otto RL Muona J McClarin J (2014) Description of *Dirrhagofarsusernae* n. sp. With a key to known *Dirrhagofarsus* species (Coleoptera: Eucnemidae). Zootaxa 3878: 179–185. 10.11646/zootaxa.3878.2.4 25544441

[B79] Paiero SM Jackson MD Jewiss-Gaines A. Kimoto T Gill BD Marshall SA (2012) Field guide to the jewel beetles (Coleoptera: Buprestidae) of northeastern North America. Canadian Food Inspection Agency Publication A104-94/2012E, 411 pp.

[B80] Parry RH (1986) The systematics and biology of the flea beetle genus *Crepidodera* Chevrolat (Coleoptera: Chrysomelidae) in America, north of Mexico. Insecta Mundi 1: 156–196.

[B81] Parsons CT (1943) A Revision of Nearctic Nitidulidae. Bulletin of the Museum of Comparative Zoology 92: 121–278.

[B82] Pelletier G Hébert C (2014) The Cantharidae of eastern Canada and northeastern United States. Canadian Journal of Arthropod Identification 25: 1–246. 10.3752/cjai.2014.25

[B83] Pelletier G Hébert C (2019) The Cryptophagidae of Canada and the northern United States of America. Canadian Journal of Arthropod Identification 40: 1–305.

[B84] Pentinsaari M Anderson R Borowiec L Bouchard P Brunke A Douglas H Smith ABT Hebert PDN (2019) DNA Barcodes reveal 63 overlooked species of Canadian beetles (Insecta, Coleoptera). ZooKeys 894: 53–150. 10.3897/zookeys.894.37862 31844409 PMC6906170

[B85] Pinto JD Bologna MA (2002) Family 111. Meloidae Gyllenhal 1810. In: Arnett RH Jr Thomas MC Skelley PE Frank JH (Eds) American Beetles.Volume 2. Polyphaga: Scarabaeoidea through Curculionidea, CRC Press, Boca Raton, Florida, 522–529.

[B86] Rabaglia RJ Cognato AI Hoebeke ER Johnson CW Labonte JR Carter ME Vlach JJ (2019) Early detection and rapid response: A 10-year summary of the USDA Forest Service program of surveillance for non-native bark and ambrosia beetles. American Entomologist 65: 29–42. 10.1093/ae/tmz015

[B87] Rabaglia RJ Dole SA Cognato AI (2006) Review of American Xyleborina (Coleoptera: Curculionidae: Scolytinae) occurring North of Mexico, with an illustrated key. Annals of the Entomological Society of America 99: 1034–1055. 10.1603/0013-8746(2006)99[1034:ROAXCC]2.0.CO;2

[B88] Ranger CM Reding ME Persad AB Herms DA (2010) Ability of stress-related volatiles to attract and induce attacks by *Xylosandrusgermanus* and other ambrosia beetles. Agricultural and Forest Entomology 12: 177–185. 10.1111/j.1461-9563.2009.00469.x

[B89] Riley EG Clark SM Flowers RW Gilbert AJ (2002) Family 124. Chrysomelidae Latreille 1802. In: Arnett RH Jr Thomas MC Skelley PE Frank JH (Eds) American Beetles.Volume 2. Polyphaga: Scarabaeoidea through Curculionidea, CRC Press, Boca Raton, Florida, 617–691.

[B90] Riley EG Clark SM Seeno TN (2003) Catalog of the leaf beetles of America north of Mexico. Coleopterists Society Special Publication 1, 290 pp.

[B91] Schülke M Smetana A (2015) Staphylinidae. In: Löbl I Löbl D (Eds) Catalogue of Palaearctic Coleoptera.Volume 2/1 revised and updated. Hydrophiloidea – Staphylinoidea. Brill, Leiden and Boston, 304–900.

[B92] Seevers LH (1978) A generic and tribal revision of the North American Aleocharinae (Coleoptera: Staphylinidae) [with additions and annotations by Lee H. Herman]. Fieldania: Zoology 71, Publication 1282, vi + 289 pp. 10.5962/bhl.title.3136

[B93] Sikes DS Trumbo ST Peck SB (2016) Cryptic diversity in the New World burying beetle fauna: *Nicrophorushebes* Kirby; new status as a resurrected name (Coleoptera: Silphidae; Nicrophorinae). Arthropod Systematics & Phylogeny 74: 299–309.

[B94] Steiner WE Jr. (1984) A review of the biology of phalacrid beetles (Coleoptera). In: Wheeler Q Blackwell M (Eds) Fungus-Insect Relationships: Perspectives in Ecology and Evolution.Columbia University Press, New York, 424–455.

[B95] Steiner WE Jr (2016) New assignments among the genera *Haplandrus* LeConte, *Metaclisa* Jacquelin Du Val and *Tharsus* LeConte with descriptions of larvae and pupae and a new genus for North America (Coleoptera: Tenebrionidae). Annales Zoologici 66: 529–550. 10.3161/00034541ANZ2016.66.4.005

[B96] Steinhauer AL (1959) The biology and seasonal development of the vetch bruchid, *Bruchusbrachialis* Fåhraeus, in Oregon. Journal of Economic Entomology 52: 955–957. 10.1093/jee/52.5.955

[B97] Tuttle DM (1954) Notes on the bionomics of six species of *Apion* (Curculionidae, Coleoptera). Annals of the Entomological Society of America 47: 301–307. 10.1093/aesa/47.2.301

[B98] Vaurie P (1948) A review of the North American Languriidae. Bulletin of the American Museum of Natural History 92: 119–156.

[B99] Van Vondel BJ Alarie Y (2016) A new species of *Haliplus* Latreille, 1802 (Coleoptera: Adephaga: Haliplidae) from Canada. The Coleopterists Bulletin 70: 801–804. 10.1649/0010-065X-70.4.801

[B100] Wagner JA (1975) Review of the genera *Euplectus*, *Pycnoplectus*, *Leptoplectus*, and *Acolonia* (Coleoptera: Pselaphidae) including Nearctic species north of Mexico. Entomologica Americana 49: 125–207.

[B101] Wappes JM Santos-Silva A (2019) A new species and synonymy in North American Phymatodes (Phymatodes) Mulsant, 1839 (Coleoptera: Cerambycidae: Cerambycinae: Callidiini). Insecta Mundi 0687: 1–9.

[B102] Watrous L (1980) Lathrobium (Tetartopeus): natural history, phylogeny and revision of the Nearctic species (Coleoptera, Staphylinidae). Systematic Entomology 5: 303–338. 10.1111/j.1365-3113.1980.tb00418.x

[B103] Weber BC McPherson JE (1983) World list of host plants of *Xylosandrusgermanus* (Blandford) (Coleoptera: Scolytidae). The Coleopterists Bulletin 37: 114–134.

[B104] Webster RP (2016) Checklist of the Coleoptera of New Brunswick, Canada. In: Webster RP Bouchard P Klimaszewski J (Eds) The Coleoptera of New Brunswick and Canada: providing baseline biodiversity and natural history data.ZooKeys 573: 387–512. 10.3897/zookeys.573.8022 PMC4829933 27110174

[B105] Webster RP Alderson CA Webster VL Hughes CC Sweeney JD (2016a) Further contributions to the longhorn beetle (Coleoptera, Cerambycidae) fauna of New Brunswick and Nova Scotia, Canada. ZooKeys 552: 109–122. 10.3897/zookeys.552.6039 PMC4740852 26865818

[B106] Webster RP Anderson RS Sweeney JD DeMerchant I (2012a) New Coleoptera records from New Brunswick, Canada: Anthribidae, Brentidae, Dryophthoridae, Brachyceridae, and Curculionidae, with additions to the fauna of Quebec, Nova Scotia and Prince Edward Island. In: Anderson R Klimaszewski J (Eds) Biodiversity and Ecology of the Coleoptera of New Brunswick, Canada.ZooKeys 179: 349–406. 10.3897/zookeys.179.2626 PMC3337068 22539901

[B107] Webster RP Davies AE Klimaszewski J Bourdon C (2016b) Further contributions to the staphylinid fauna of New Brunswick, Canada, and the USA, with descriptions of two new *Proteinus* species (Coleoptera, Staphylinidae). In: Webster RP Bouchard P Klimaszewski J (Eds) The Coleoptera of New Brunswick and Canada: providing baseline biodiversity and natural history data.ZooKeys 573: 31–83. 10.3897/zookeys.573.7830 PMC4829926 27110167

[B108] Webster RP Klimaszewski J Pelletier G Savard K (2009) New Staphylinidae (Coleoptera) records with new collection data from New Brunswick, Canada. I. Aleocharinae. In: Majka CG Klimaszewski J (Eds) Biodiversity, ecology of Canadian Coleoptera II.ZooKeys 22: 171–248. 10.3897/zookeys.22.152

[B109] Webster RP Sweeney JD DeMerchant I (2012b) New Coleoptera records from New Brunswick, Canada: Sphindidae, Erotylidae, Monotomidae, and Cryptophagidae. In: Anderson R Klimaszewski J (Eds) Biodiversity and ecology of the Coleoptera of New Brunswick, Canada.ZooKeys 179: 169–192. 10.3897/zookeys.179.2466 PMC3337060 22539893

[B110] Webster RP Sweeney JD DeMerchant I (2012c) New Coleoptera records from New Brunswick, Canada: Geotrupidae and Scarabaeidae. In: Anderson R Klimaszewski J (Eds) Biodiversity and ecology of the Coleoptera of New Brunswick, Canada.ZooKeys 179: 27–40. 10.3897/zookeys.179.2607 PMC3337050 22539883

[B111] Webster RP Sweeney JD DeMerchant I (2012d) New Coleoptera records for New Brunswick, Canada: Kateretidae, Nitidulidae, Cerylonidae, Endomychidae, Coccinellidae, and Latridiidae. In: Anderson R Klimaszewski J (Eds) Biodiversity and ecology of the Coleoptera of New Brunswick, Canada.ZooKeys 179: 193–214. 10.3897/zookeys.179.2581 PMC3337061 22539894

[B112] Webster RP Sweeney JD DeMerchant I (2012e) New Staphylinidae (Coleoptera) records with new collection data from New Brunswick, Canada: Scaphidiinae, Piestinae, Osorinae, and Oxytelinae. In: Klimaszewski J Anderson R (Eds) Biosystematics and Ecology of Canadian Staphylinidae (Coleoptera) II.ZooKeys 186: 239–262. 10.3897/zookeys.186.2506 PMC3349196 22577322

[B113] Webster RP Webster VL Alderson CA Hughes CC Sweeney JD (2016c) Further contributions to the Coleoptera fauna of New Brunswick with an addition to the fauna of Nova Scotia, Canada. In: Webster RP Bouchard P Klimaszewski J (Eds) The Coleoptera of New Brunswick and Canada: providing baseline biodiversity and natural history data.ZooKeys 573: 265–338. 10.3897/zookeys.573.7327 PMC4829930 27110171

[B114] Wood SL (1992) Nomenclatural changes and new species in Platypodidae and Scolytidae (Coleoptera), Part II. Great Basin Naturalist 52: 78–88.

[B115] Woodroffe GE Coombs CW (1961) A revision of the North American *Cryptophagus* Herbst (Coleoptera: Cryptophagidae). Miscellaneous Publications of the Entomological Society of America 2: 179–211.

